# The effects of policy actions to improve population dietary patterns and prevent diet-related non-communicable diseases: scoping review

**DOI:** 10.1038/ejcn.2016.234

**Published:** 2016-11-30

**Authors:** L Hyseni, M Atkinson, H Bromley, L Orton, F Lloyd-Williams, R McGill, S Capewell

**Affiliations:** 1 Department of Public Health and Policy, University of Liverpool, Liverpool, UK

## Abstract

Poor diet generates a bigger non-communicable disease (NCD) burden than tobacco, alcohol and physical inactivity combined. We reviewed the potential effectiveness of policy actions to improve healthy food consumption and thus prevent NCDs. This scoping review focused on systematic and non-systematic reviews and categorised data using a seven-part framework: price, promotion, provision, composition, labelling, supply chain, trade/investment and multi-component interventions. We screened 1805 candidate publications and included 58 systematic and non-systematic reviews. Multi-component and price interventions appeared consistently powerful in improving healthy eating. Reformulation to reduce industrial trans fat intake also seemed very effective. Evidence on food supply chain, trade and investment studies was limited and merits further research. Food labelling and restrictions on provision or marketing of unhealthy foods were generally less effective with uncertain sustainability. Increasingly strong evidence is highlighting potentially powerful policies to improve diet and thus prevent NCDs, notably multi-component interventions, taxes, subsidies, elimination and perhaps trade agreements. The implications for policy makers are becoming clearer.

## Introduction

Non-communicable diseases (NCDs) now exceed 34 million deaths annually (65% of global deaths). NCDs, particularly cardiovascular diseases, common cancers, respiratory diseases, diabetes and dementia, are a common cause of disability.^1^

Four major risk factors generate the majority of NCDs. Of these, poor diet makes the biggest contribution, larger than tobacco, alcohol and physical inactivity combined.^2^ Globally, increased consumption of processed food containing excess amounts of sugar, salt, saturated and trans fats are compounded by low intakes of healthy foods like fruit and vegetables, whole grains, nuts, pulses and seafood.

Hence a key policy question is: how can diets be improved to prevent NCDs? Two options are often discussed: ‘downstream' behavioural interventions focusing on individuals or, the focus of this review, ‘upstream' policy interventions that aim to promote healthier environments.^3^ Worldwide, many different ‘upstream' policy options have been proposed to promote healthier eating and prevent diet-related NCDs. These include targeting price, promotion, provision, composition, labelling, supply chain, trade and investment, or a combination thereof.^4^ However, these options are broad and potentially confusing, with continuing uncertainties about which interventions are most effective. To inform their decisions, policy makers need clear evidence on the potential population benefits (as well as the ease and cost of implementation).

This scoping review, therefore, collates the current evidence on policy interventions to promote healthier dietary consumption.

## Methods

### Search strategy

We first identified exemplar studies of dietary interventions, which were used to define and refine search terms. This process identified interventions targeting price, promotion, provision, composition, labelling, supply chain, trade and investment.^4^ This search strategy was piloted for price interventions to determine appropriate databases for identifying all relevant studies and was adapted for the remaining interventions.

The search strategy consisted of a combination of two sets of keywords. The first one focused on dietary components such as salt, fruit, vegetables, fat, trans fat, sugar, diet or equivalents of these terms. The second search string focused on policy interventions including taxes, subsidies, reformulation, elimination, labelling, campaigns, marketing, advertisement, workplace, schools, community, food system, food supply chain, food trade, food investment, legislation, regulation or equivalents of these terms.

A total of six databases were searched: MEDLINE; Science Citation Index, SCOPUS, Cochrane Database of Systematic Reviews, The Campbell Collaboration Library of Systematic Reviews and the CRD Wider Public Health database. These were identified during the pilot searches as the most appropriate databases to generate relevant papers on this specific topic. Reference lists of included papers were also scanned for potential eligible reviews and systematic reviews (SR).

### Selection of studies and inclusion criteria

Systematic and non-SRs addressing policy interventions to improve healthy eating and to prevent NCDs, with a quantitative outcome, dating from January 2004 onwards were included. A PICOS approach (participants, interventions, comparators, outcomes and study design) was used to assess the retrieved studies (Table 1). Only reviews in English were considered.

One researcher (LH) conducted the searches and removed duplicates. All remaining titles and abstracts were screened independently by two researchers (LH and MA), based on the inclusion/exclusion criteria (Table 1). All papers deemed potentially eligible were retrieved in full-text form and again screened independently by two researchers. Discrepancies on whether to include or exclude reviews were resolved by consensus or by seeking the opinion of a senior team member (SC).

### Data extraction

Both empirical studies (presenting primary data) and modelling studies (using secondary data to project health outcomes) were included. However, the results were analysed and discussed separately, recognising their different natures.

Data extraction was done by one researcher using pre-designed and pre-piloted forms (Table 2 and Supplementary file 1). Extracted data was independently verified by another researcher to ensure accuracy and completeness. Owing to logistical and time constraints, it was not possible to contact study authors regarding any unclear, missing or additional data.

### Data categorisation

Evidence from the reviews was categorised using a seven-part framework, which was developed and refined from the ‘4Ps' marketing framework:^4^ price, place, promotion and product. In social marketing these 4Ps are key in planning and implementing strategies and this translates well to a policy context. In the social marketing mix, product stands for the desired behaviour and what people will gain when they perform the behaviour, whereas in the policy context this refers to the actual product (for example, foods). Price refers to the cost exchanged for the promised benefits and place refers to the location where the goods are distributed. Finally, promotion refers to a persuasive communication strategy to influence change and can involve multiple channels and target groups. After extensive piloting the following categories were used:

- Food price—policies influencing prices through taxes, subsidies or economic incentives;
- Food promotion—advertising/marketing; particularly on children; media campaigns and health education;
- Food provision—in specific settings: schools, communities or workplaces;
- Food composition—reformulation or elimination;
- Food labelling—nutrition labelling, calorie labelling in stores/restaurants;
- Food supply chain, trade and investment—including legislation or regulation affecting production policies or supply-chain logistics;
- Multi-component interventions (including at least two of the categories described above).

### Data analysis

We used a narrative synthesis to summarise the evidence of policy interventions to promote healthy eating patterns.^55^ First, the data identified and included in this review were extracted in tables and grouped based on their type of intervention. The different types of interventions included are discussed under data categorisation and are adapted from the 4P's marketing framework. Most interventions have subcategories, for example, food provision, which can be done in schools, communities or at workplaces. Within these categories the data were grouped according to the subcategories. Furthermore, as this review is about healthy eating, multiple nutrients are included. So another layer was added for these nutrients and the results were presented under their specific (sub-) categories for each of the nutrients. This structure was then used to present the results.

All the evidence for each sub-category was reviewed and analysed according to study design and the number of studies included investigating the same intervention and outcome. The key findings are presented in the results section and all the studies are then summarised in the tables.

As the study designs and evidence per nutrition varies greatly no quantitative comparison will be made between the interventions. They will each be presented separately.

## Results

**Figure 1:**
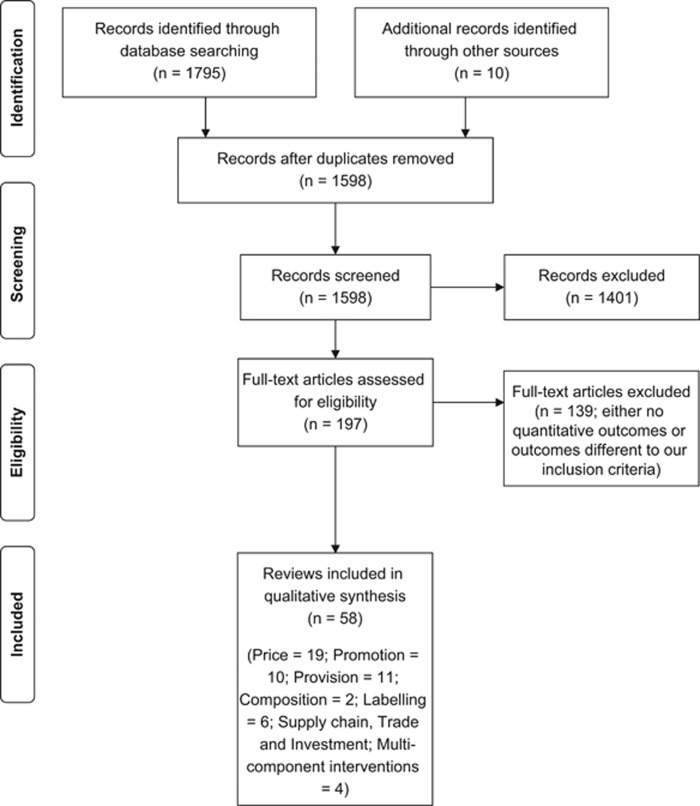
PRISMA flow diagram.

About 1805 candidate publications were screened and 197 papers were retrieved for full-text screening. In all 139 were excluded as they had no quantitative outcomes or outcomes different to our inclusion criteria. We identified 58 systematic and non-SRs for inclusion (Figure 1). Eight reviews provided information on more than one category.

### Food price

#### Taxes

One SR estimated a mean price (Table 2a) elasticity of −0.8 to −1.0 for sugar-sweetened beverages (SSBs). Thus, a 10% price increase is associated with an 8–10% reduction in SSB consumption.^16^ Furthermore, Thow *et al.*^22^ reported a 5–48% reduction in SSB consumption proportional to the modelled taxes applied (5–30%).

However, taxing unhealthy food may cause substitution effects. For instance, a fat tax might reduce saturated fat content but increase salt content and product consumption.^23^ Four modelling studies included in Thow *et al.*^22^ investigated these substitution effects. A 5–20% SSB tax could reduce calorie intake of SSBs by 10–48% in adults and by 5–8% in children, however consumption of milk, tea, coffee and low-calorie beverages may increase correspondingly.^22^ Finally, Welsh *et al.*^7^ included a modelling study suggesting that a US penny-per-ounce tax might reduce SSB consumption by 15% among adults and subsequently prevent ~95 000 coronary heart events, ~8000 strokes and ~26 000 premature deaths.

Several reviews included at least one modelling study investigating a ‘fat tax'.^9, 10, 19, 22^ All suggested that changes in fat consumption would be minor, unless the tax was substantial. Four modelling studies in Thow *et al.*^22^ suggested that a small fat tax (5–17%) might reduce (saturated) fat consumption by 0–3% and induced substitution with lower-fat options. Increasing the price of salty foods by 40% was suggested to reduce sodium consumption by ~6%.^22^

Mozaffarian *et al.*^20^ included a modelling study investigating a combination of taxing less healthy foods and using the $580 million revenue raised to subsidise fruit and vegetables. This might alter consumption to prevent ~6000 CVD and cancer deaths annually.

#### Subsidies

Powell *et al.* included six modelling studies reporting −0.49 mean price elasticity for fruit and vegetables, suggesting a 20% subsidy on fruit and vegetables might increase consumption by 10%,^18^ while a 10% subsidy might increase consumption by 5%.^22^ A continuing price reduction on fruit and vegetables might prevent ~6700 cases of CHD and 2950 cases of ischaemic stroke.^21^

### Food promotion

#### Marketing and advertising

Four randomised controlled trials (RCTs) in Gregori *et al.*^25^ (Table 2b) indicated that exposure to food advertisements compared with non-food advertisements increased calorie intake and snacks consumed. The effect appeared largest in obese children. Two cross-sectional studies by Boyland and Halford^26^ supported these findings. Conversely, banning food advertisements on television might reduce the US baseline rate of childhood obesity by 2.5–6.5%.^27^

#### Nutrition education

RCTs reviewed in two SRs suggested that nutrition education focused on fruit and vegetables could increase their intake by 0.4–1.4 servings per day.^28, 30^ Two additional RCTs reported a net increase of 0.4 servings per day.^6^

A meta-analysis of RCTs found that e-learning interventions focused on dietary intake increased fruit and vegetable intake by 0.24 servings per day; reduced total fat (−0.8 g), saturated fat (−0.2 g) and daily energy intake (4 kcal).^29^

#### Media campaigns

Three reviews focused on fruit and vegetables.^31, 32, 33^ Benefits were modest at best.

### Food provision in schools, communities or workplaces

#### Schools

A cross-sectional and longitudinal study included in Levy *et al.*^6^ (Table 2c) investigated the effect of eliminating access to SSBs on consumption and found reductions of 0.16 servings per day and 4%, respectively. Another time-series study found a 35% decline in consumption in schools after SSBs were removed from cafeteria vending machines. However, pupils obtained SSBs from home or non-cafeteria vending machines instead.^6^ A modelling study by Patel and Cabana^36^ suggested replacing SSBs with water might reduce intake by 235 kcal per day among 2–19 year olds.

Jaime and Lock^38^ reviewed the effect of a free piece of fruit in schools in three RCTs. Fruit and vegetable intake increased by 0.30–0.44 servings per day. However, benefits were not necessarily sustained long term.^38^ Fat intake was targeted by one RCT and two non-randomised studies in the same review, reporting decreases in saturated fat (0.9–5.2% of energy intake) and total fat (2.0–10.9%).^38^ A meta-analysis of school interventions targeting fruit and vegetable intake found a higher effect when fruit juice was included (0.32 portions) compared with no fruit juice included (0.25 portions).^37^

#### Communities

No reviews were found for community interventions and the effect on dietary intake or health outcomes.

#### Workplaces

Thompson and Ravia^30^ reviewed RCTs of work-based interventions and reported 0.8 servings per day increase in fruit and vegetable intake. A meta-analysis of 36 RCTs also found a significant increase in daily fruit and vegetable consumption.^35^ Five RCTs in Mhurchu *et al.*^34^ focused on total fat intake as a percentage of energy and found reductions in both the intervention (−2.2 to −9.1%) and control groups (+1.3 to −1.8%).

### Food composition

#### Trans fats

Downs *et al.*^42^ included two pre–post-test studies evaluating (Table 2d) the effectiveness of interventions aimed to reduce trans fatty acids (TFAs) in food. In Denmark average iTFA intake decreased from 4.5 g per day in 1976 to 1.5 g per day in 1995 and reduced to virtually zero after the ban in 2005.^34^ In The Netherlands, voluntary reformulation of TFAs decreased daily energy intake from 1.0 to 0.8% (a 20% reduction).^42^

Mozaffarian and Clarke^43^ conducted two meta-analyses with 13 RCTs and 4 prospective studies to calculate the effect of reduced TFAs in partially hydrogenated vegetable oils by means of reformulation. The predicted risk reductions were greatest when replacing at least half of the iTFA in partially hydrogenated vegetable oils, with soybean oil or canola oil.^43^

### Food labelling

#### Menu labelling

Two SRs evaluated the effect of menu labelling on dietary intake (Table 2e). Swartz *et al.*^46^ included two non-blind RCTs that reported no significant impact of menu labelling on calorie consumption. However, when combining the labels and labels/information categories vs no label, the labelled condition consumed 180 fewer calories.^46^ Similar results were found by a meta-analysis of 17 studies conducted by Sinclair *et al.*^45^

#### Nutrition labelling

Observational studies in Campos *et al.*^47^ reported an association between nutrition label use and fat consumption. Furthermore, experimental research included showed that participants tended to consume more energy reduced foods in terms of weight, but the overall energy intake was significantly lower.^47^ The theoretical concern that labelling might lead to overconsumption of foods perceived as ‘less unhealthy' and appeared to be unfounded in a cross-sectional study by Hawley *et al.*^49^

### Food supply chain, trade and investment

#### Trade and market liberalisation

Evidence for the effect of trade (Table 2f) market liberalisation on dietary intake is contested. Traill suggested only ‘modest' effects,^52^ whereas a study included in Friel *et al.*^51^ argues that liberalisation of Foreign Direct Investment through trade agreements with the USA significantly increased soft drink consumption within low- and middle-income countries.

#### Monetary subsidies and taxes

Thow *et al.*^21^ included two modelling studies; one suggested that increasing value-added tax in the UK on the main sources of saturated fat might reduce consumption and thereby decrease CHD deaths by 1.8–2.6%. The other suggested that halving value-added tax on fruit and vegetables could decrease consumption of sugar (−6.5%), fat (−2.5%) and saturated fat (−3.6%).^21^

#### Reformulation

Livingstone *et al.*^44^ reviewed reformulation in the supply chain by altering the diet of cows and investigated the effect on milk and dairy products. A double-blinded randomised cross-over intervention study found a 16% decrease in saturated fats and increase in monounsaturated fats (+10%) and polyunsaturated fats (+7.5%). A meta-analysis of RCTs suggested that replacing 10% of dietary saturated fat intake with polyunsaturated fats might reduce CHD risk by 10%.^44^

### Multi-component interventions

#### Trans fats

Voluntary limits combined with mandatory labelling (Table 2g) were associated with a 30% reduction in dietary trans fat intake in the general population, in an interrupted time-series study in Downs *et al.*^42^

#### Fruit and vegetables

Fifteen intervention studies in Blanchette and Burg^54^ combined provision and promotion activities, and found an increase in fruit and vegetable intake of 2.54 servings per day. Conversely, single interventions rarely increased intake by more than 0.5 servings.^38, 39, 40^

#### Salt

No reviews have evaluated the effects of multi-component interventions on salt intake. However, the UK salt programme has been evaluated and a 1.5 g per day (15%) reduction was observed between 2001 and 2011. This was achieved by sustained and progressive industry reformulation involving close monitoring and political pressure to ensure compliance, reinforced by media campaigns and traffic light food labelling.^56, 57^

## Discussion

### Main findings

Our rapid scoping review suggests that ‘upstream' interventions such as price interventions appear to be consistently effective in improving healthy eating.^9, 16, 18, 19, 20, 21, 25^ Multi-component interventions and reformulation also appear effective in promoting healthy diets. However, the effectiveness of the remaining policy interventions (labelling, restrictions on the provision or marketing of unhealthy food) generally demonstrated smaller effects and less certain long-term benefits.^29, 36^

### Specific findings

#### Food price

Taxes and subsidies appear to be consistently effective at reducing consumption of sugary drinks,^16, 22^ and increasing the consumption of fruit and vegetables.^18, 21, 22^ Changes in consumption demonstrated price elasticity, thus the larger the taxes or subsidies, the greater the effect.^16, 22^ A fat tax, however, appeared to have smaller impacts on consumption,^9, 19, 22^ and potential substitution effects need to be anticipated and negated.^22, 23^

#### Food promotion

Junk food advertisements significantly increase total calorie intake. Banning advertising to children therefore appears to offer an effective intervention to decrease intake and potentially reduce obesity levels.^25, 26^ Health promotion campaigns^31, 32, 33^ and nutrition education^6, 28, 29, 30^ showed small effect sizes and benefits may diminish over time. More studies are needed to determine long-term effects.

#### Food provision

Most school-based interventions focused on fruit and vegetables^37, 38^ or access to SSBs.^6, 36^ Overall, these interventions seemed to be effective in the short term but effect sizes are generally small and longer-term effects were not routinely investigated.

The majority of workplace interventions focused on fruit and vegetable intake,^30, 35^ with a few focusing on fat intake,^34^ and effects were generally modest.

#### Food composition

Mandatory reformulation to eliminate trans fat was very effective in Denmark, reducing consumption far more than voluntary reformulation. This is in line with a modelling study comparing voluntary with mandatory salt reformulation policies.^58^ Furthermore, Cappuccio *et al.*^59^ suggested that nation-wide reformulation policies might reduce salt consumption by 1 g per day.

#### Food labelling

Although menu labelling appears weak or ineffective, food labelling may help consumers to choose and purchase healthier options,^46^ while also exerting pressure on manufacturers to reformulate.^60^ Furthermore, although 50% of EU consumers might use food labels; many report difficulties in interpreting the labels.^47, 61^ However, the current evidence is based on diverse methodologies and remains limited.^62^

#### Food supply chain, trade and investment

Food supply chain interventions appear intuitively powerful, but are under-researched. Interventions could take place at many levels of the food supply chain, with positive (or negative) effects on food availability, price, quality or marketing.^51, 52, 53^ Targeted reformulation, subsidies and taxes in the food supply chain could have beneficial effects on diet-related health outcomes.^44^

#### Multi-component interventions

Multi-component interventions appear to be highly effective at reducing the dietary intake of salt, industrial trans fats and increasing fruit and vegetable consumption, thus improving health outcomes at population level.^56, 57^ However, salt intake remains very high in most countries; often double the World Health Organisation recommended maximum levels of 5 g per day.^63^

Likewise, industrial trans fat levels have been slashed in Denmark, the USA and parts of Western Europe but remain high elsewhere.^42^ Furthermore, the reviews included only considered trans fat and fruit and vegetable intake. It is, therefore, not clear whether multi-component interventions might be equally effective for other dietary components such as sugar and total fat.

### Strengths of this analysis

This scoping review used two independent researchers to screen all the papers generated through the searches. Discrepancies were resolved through consensus or consulting a third senior researcher. Furthermore, a systematic approach was used based on the seven-item interventions framework.

Food supply chain, trade and investment is an underexplored area; however there is some evidence to suggest that globalisation and liberalisation of markets can influence dietary patterns and NCDs. Multi-component interventions are as suggested effective to reduce dietary intake and NCDs. Countries have implemented several policies at the same time and it is important to quantify the impact compared with single policies.

### Limitations of this analysis

This scoping review has several limitations. First, we only searched for reviews from the last 10 years. However, we anticipate this captured majority of relevant reviews and thus provide an excellent overview of current food policy interventions. Second, only reviews with an abstract available in English were included. Third, this scoping review focussed on systematic and non-SRs only, thereby missing areas lacking such reviews. However, we explicitly included both systematic and non-SRs to explore under-researched areas. Fourth, some effect measures were too unclear to include in the tables because it was not possible to go back to individual studies. Furthermore, as our inclusion criteria were only focused on dietary intake (consumption) and health outcomes; studies considering any other precursors to dietary behaviour such as purchasing behaviour, awareness, knowledge, preferences and availability were not presented. Also, most studies included were conducted in high-income or middle-income countries; generalisation of the results to low-income settings might be problematic. Fifth, whereas the effects of price interventions can be quantified and occurred immediately, nutrition education and health promotion campaigns have long-term effects that cannot be so easily measured. Finally, systematic and non-SRs included many different study methodologies. Outputs from RCTs, observational cohorts, natural experiments and modelling studies are all potentially valid, but may not be directly comparable and should therefore be interpreted with caution. We have, therefore, routinely presented such results separately.

### Future research

The logical next step is to conduct a fully resourced SR that includes all relevant primary studies. This could explore whether effectiveness varies in different ethnic or socioeconomic sub-groups, as socioeconomic deprivation is often associated with increased intake of foods high in saturated fat and sugar, poor nutrition and poorer health. Downstream interventions (such as advice to individuals) may widen the inequalities gap, whereas population-based policy interventions may reduce inequalities. Finally, the few analyses of multi-component interventions suggest that they may be more effective than single interventions. Further research is needed in all these areas.

## Conclusions

Diverse policy interventions exist to improve healthy eating and thus prevent NCD. The evidence base varies for each category and nutrient. The evidence base for price interventions appears to be comprehensive and included studies mostly on taxing SSBs, followed by taxing dietary fat. Studies investigating the effect of subsidies mainly focused on fruit and vegetable intake. Taxes consistently decrease SSB consumption, whereas subsidies increase fruit and vegetable intake. Only a few reviews were included for food composition. The Denmark trans fat story suggests that voluntary reformulation can be effective to start with; however, a legislative ban is able to then essentially eliminate industrial trans fat consumption. Mandatory reformulation is thus consistently more effective than voluntary reformulation. Health promotion campaigns and nutrition education benefits appear modest and effects usually reduce over time. Limiting the marketing of junk food and sugary drinks appears to be effective and, thus, a ripe target for regulation.

Workplace interventions appeared to be modestly effective at increasing fruit and vegetable intake. School interventions showed a modest effect of reducing SSB consumption and increase fruit and vegetable consumption in schools. The effects for food labelling were mixed. Labelling may help pressure reformulation by manufacturers and inform consumer choice. However, much depends on individual awareness, knowledge and ability to interpret labels correctly. Trade and investment factors controlling the food supply chain may be powerful but are currently under-researched. Finally, multi-component interventions appear to be more effective than single interventions. This might be predicted, having previously been reported in tobacco control, where comprehensive, coordinated programs are superior to single interventions.^64^

## Figures and Tables

**Table 1:** Inclusion/exclusion criteria

*Participants*	
*Include*	*Exclude*
Systematic and non-systematic reviews reporting a quantitative assessment of the effects (intended or unintended) of actions to improve healthy eating	All studies considered ineligible

*Interventions*	
Systematic and non-systematic reviews evaluating the effects of dietary actions that are: implemented by government policy; applied experimentally; or adopted in specific settings	

*Comparators*	
Systematic and non-systematic reviews were included in the review where actions to improve healthy diet and actions promoting NCD prevention were evaluated or compared	Reviews were excluded if no comparisons of different actions to improve healthy diet or preventing NCDs were presented

*Outcomes*	
The primary outcome of interest was dietary intake (consumption) Secondary outcomes included changes in clinical/physiological indicators related to NCDs and behaviours associated with a healthy diet	Process evaluations reporting on implementation of interventions or policies without any quantitative outcome data; data only on costs, feasibility or acceptability without an assessment of primary effects (intake); studies on individuals as opposed to groups or whole populations; under nutrition; micro-nutrient deficiencies, micro-nutrient fortification; supplements

*Study design*	
Systematic and non-systematic reviews that consisted of modelling studies, secondary analyses, randomised controlled trials, natural experiments, before vs after interventions and modelling studies were included. Furthermore, this review included studies for all age groups and all populations from high-, middle- and low-income countries	Commentary/opinion articles; purely qualitative evaluations with no quantitative assessment; data/statistics from monitoring and surveillance that were not directly linked to a policy intervention; reviews/studies of under nutrition

Abbreviation: NCD, non-communicable disease.

**Table 2:** Summary tables

	*Study*	*Study type*	*Geographical scope*	*Aim and main outcomes*	*Relevant results*
**(a) Food price**					
Taxes (SSBs)	Cabrera Escobar *et al.*^5^	Systematic review and meta-analysis of cross-sectional and longitudinal studies	USA, Brazil, Mexico and France	Aim: to evaluate the literature on SSB taxes or price increases Outcomes: consumption and demand	SSB price elasticity (9 studies): pooled own price elasticity was −1.299 (95% CI: −1.089 to −1.509). Cross-price elasticities (four studies): four articles reported cross-price elasticities, three from the USA and one from Mexico; higher prices for SSBs were associated with an increased demand for alternative beverages such as fruit juice (0.388, 95% CI: 0.009–0.767) and milk (0.129, 95% CI: −0.085 to 0.342), and a reduced demand for diet drinks (−0.423, 95% CI: −0.628 to −1.21). Higher prices for SSBs were associated with an increased demand for alternative beverages such as fruit juice and milk, and a reduced demand for diet drinks. One cross-sectional study: a 1% increase in SSB price, the point prevalence for obesity would reduce more in men (−0.34 percentage points) than in women (−0.05) One longitudinal study: found a reduction in the point prevalence of obesity (−0.03 percentage points) in adults
	Levy *et al.*^6^	Review of RCTs and population studies	USA, UK, Belgium, Brazil, Canada and Holland	Aim: to review the literature on school nutrition policies and price interventions Outcomes: SSB consumption	Effect on kcal (two population studies): a 1% increase in the tax rate was associated with nearly 8 fewer kcal from soda consumed (*P*<0.05), a ~6% reduction; whereas a 20% SSB price increase was found to reduce SSB energy intake by 38.8% for adults and 48.8% for youth. Effect on consumption (review): the price elasticity for soft drinks is in the range of −0.8 to −1.0 meaning that a 10% price increase was associated with an 8% reduction in soda consumption. Effect on consumption and kcal (review): a tax increase at 20% of current prices would reduce consumption by between 16 and 20% (−36 to −45 kcal, assuming average intake of 225 kcal). Effect on obesity (two population studies): no differences were found in obesity rates between states with and without a >5% tax. A 10% increase in the price of sugar products was associated with a decrease in the prevalence of obesity by 8%
	Welsh *et al.*^7^	Review of modelling studies	USA	Aim and outcomes: to present recent data on SSB consumption and its health impact	Modelling study (1): the penny-per-ounce tax would reduce the consumption of SSBs by 15% among adults and prevent 95 000 coronary heart events, 8000 strokes and 26 000 premature deaths
Taxes (Fat)	Cash and Lacanilao^8^	Review of modelling studies		Aim: to review the economic evidence of taxes Outcomes: consumption and health	Modelling study (1): the results of a possible fat tax show that an increase of 0.4–1% would not significantly affect consumption or health outcomes
	Galizzi^9^	Review of modelling studies	USA and UK	Aim: to assess the relative effectiveness of taxes Outcomes: consumption and health	Modelling study (1): a tax proportional to fat content can reduce fat intake of 1%, with a 10 times higher burden on poor consumers
	Tiffin and Salois^10^	Review	Focus on UK	Aim: to investigate the effect of nutrition policies including a fat tax Outcomes: consumption	Modelling study (1): a fat tax according to the % fat content (1.56% fat will have a tax of 1.56%), with a ceiling of 15% lead to only small changes in intakes (total fat~−1.6% saturated fat~−2.0%)
Taxes (fat and sugar)	Faulkner *et al.*^11^	Scoping review of observational studies and RCTs	USA mostly with a focus on Canada	Aim: to synthesise existing evidence regarding economic policies Outcomes: obesity	Cross-sectional study (1): states without a soft drink or snack food tax were four times more likely (statistically insignificant) than states with a tax to exhibit a high relative increase in obesity prevalence
Tax (high-energy dense foods)	Epstein *et al.*^12^	Review of experimental research	Not mentioned	Aim: to review experimental research on policy decisions Outcomes: dietary intake and health	Experimental study (1): a 50% price increase for high-energy dense foods in a lab setting resulted in a reduction in calories of 16%
Tax (sugar)	Powell and Chaloupka^13^	Review of cross-sectional and longitudinal studies	USA	Aim: to assess the relationship between food price and weight Outcomes: obesity	Tax/price increase (one longitudinal study): a one dollar increase in the current price of sugar was associated with a 0.33 percentage point reduction in the probability of obesity
Subsidies (fruit and vegetables)	Black *et al.*^14^	Systematic review of RCTs, CBAs and ITS studies	USA, UK and New Zealand	Aim: to determine the impact of the food subsidy programs Outcomes: fruit and vegetable intake	RCT (1): overweight women who received $10 per week of F&V for 6 months increased mean consumption of fruit by 1.0 serves per day (95% CI: 0.1–1.9, *P*=0.02) and vegetables by 0.9 serves per day (95% CI: 0.3–1.5, *P*=0.002) whereas the intake of controls was unchanged
	An^15^	Review of RCTs, cohort and pre–post-test studies	USA, Germany, UK, Canada, South Africa, France and Holland	Aim: to review the effectiveness of monetary subsidies Outcomes: consumption	Cohort study (1): a 10 and 25% discounts on healthier foods were associated with an increase in daily F/V intake by 0.38 and 0.64 servings, respectively. Before and after study(1): preliminary evidence shows that the demands for fruits are price elastic— a 1% decrease in price is associated with a larger than 1% increase in quantity demanded. RCT (1): when the price in hospital cafeterias was lowered on healthy foods by 15–25%, fruit intake increased by about 30%.
Price elasticities	Andreyeva *et al.*^16^	Systematic review of ITS, household surveys and retail scanner data	USA	Aim: to assess mean elasticities by food category Outcomes: consumption	Based on 160 studies: the mean price elasticity for the soft drink category (0.79, absolute value) is based on 14 estimates, with a range of 0.13–3.18 and was considered to be most elastic followed by juice, fruit, sugar and sweets, fats and oils. A 10% increase in soft drink prices should reduce consumption by 8–10%
	Maniadakis *et al.*^17^	Systematic review of longitudinal, modelling, cohort, demand and cross-sectional studies	USA, Norway and Singapore	Aim: to assess the effect of taxation Outcomes: consumption and caloric intake	Demand studies (12): the price elasticity of demand for beverages is in the range of −0.5 to −1.6, depending on the beverage. The caloric effect of a 10% increase in prices reduces energy intake by a max of 50 calories per day, which is not considered significant. Longitudinal studies (2): the price elasticity of demand for beverages and foods is in the range of −0.05 to −0.35 depending on the beverage and food considered
	Powell *et al.*^18^	Systematic review of modelling studies using time series or longitudinal data	USA	Aim: to assess the price elasticity of demand for SSBs and fruits and vegetables Outcomes: SSB and fruit and vegetable consumption	SSBs (14): mean price elasticity estimate of −1.21 implies that a tax on SSBs of 20% would reduce overall consumption by 24%. Fruit and vegetables (6): the mean price elasticity was −0.49 and −0.48, respectively, suggesting that subsidising fruits and vegetables by 20% would increase consumption by 10%
Taxes and subsidies	Eyles *et al.*^19^	Systematic review of simulation studies	The 34 member countries of the OECD	Aims: to investigate the estimated association between food pricing and intake or health outcomes Outcomes: food intakes	Carbonated soft drinks (4): the estimated mean own-PE, which represents the change in demand with a 1% change in price, for carbonated soft drinks was −0.93 (range,−0.06, −2.43) Saturated fat (3): for a flat rate tax, the mean modelled reduction in saturated fat intake in response to a 1% increase in price was −0.02% energy from saturated fat Fruit and vegetables (3): the estimated own-PE was −0.35. After sensitivity analysis the own-PE is −0.54
	Mozaffarian *et al.*^20^	Systematic review and grading of evidence	USA and UK	Aim: to identify and assess the evidence for the effectiveness of population interventions Outcomes: consumption and health	Tax on SSBs (review): a 10% increase in the price of SSBs would decrease consumption by 8–10%. Fruit and vegetable subsidy (modelling; 1): a 10% price subsidy would lead low-income US consumers to increase their intake of both F/V by 2–5%. A subsidy of $10 per week in vouchers to purchase F/V for 6 months led to a significantly higher intake in intervention groups compared with the control group (7.8 vs 4.8 servings per day). Six additional months after the intervention ended, this increase was sustained (7.5 vs 4.9 servings per day). Taxes and subsidies (modelling;1): the combination of taxing less healthy foods and using this revenue ($580 million) to subsidise the price of fruits and vegetable would sufficiently alter consumption to prevent 6000 deaths attributable to CVD and cancer each year
	Thow *et al.*^21^	Systematic review of empirical and modelling studies	USA	Aim: to assess the effect of food taxes and subsidies Outcomes: diet and health status	Tax on salty snack (modelling; 1)s: a 20% tax would reduce consumption by 115–170 g per person per year, equivalent to ~830 calories. Fruit and vegetable subsidy (modelling; 1): a subsidy that would lead to a lasting price reduction of 1% would prevent 6733 cases of CHD and 2946 cases of ischaemic stroke
	Thow *et al.*^22^	Systematic review of modelling studies and two RCTs	USA, France, Holland, UK, Brazil, Finland, Sweden, Norway, New Zealand and Australia	Aim: to assess the effect of food taxes and subsidies Outcomes: fruit and vegetable, total calories, SSB, fat, sugar and sodium consumption	Subsidies: a RCT found that a subsidy of 12.5% on healthy food had no effect on consumption. Another RCT stated that a subsidy of 50% on F/V would increase consumption by 25%. Subsidies modelling: four studies stated a subsidy of 10% on F/V increases consumption by ~5%, with one study estimating a 1.5% increase in consumption in response to a 1.8% price decrease. Three modelling studies found that subsidies paired with taxes (range 10–20%) could reduce total calories by ~1%. Tax on SSBs: 16 studies modelled the effect of a 5–30% SSB tax and showed a reduction in consumption (5–48%) demonstrating that consumption was proportional to the taxes applied. Substitution effects modelling: four studies modelled substitution between beverages in response to a 5–20% tax and suggested that consumers would reduce caloric intake of SSBs, by 10–48% in adults and by 5–8% in children and increase consumption of other beverages such as milk, low-calorie beverages, tea and coffee. One study suggested that a focus on a single nutrient may increase intakes of other unhealthy nutrients. Fat tax modelling: four studies showed that relatively small taxes on fat (5–17.5% $0.005 per g) can reduce fat and/or saturated fat consumption by 0–3%, reduce consumption of certain high-fat foods, and induce substitution with lower-fat options. Sugar subsidy modelling (1): an implicit sugar subsidy would result in a price decrease of 36% and an increase in consumption (7.5%). Sodium tax modelling (1): a sodium tax that increased the price of salty foods by 40% would reduce sodium consumption by 6%. Taxes based on nutrient profiling (8): taxes on ‘unhealthy'/‘high calorie' foods ranged from 10–50% and reduced consumption by 6.5–8%. A 25% tax on ‘red' labelled foods significantly reduced consumption among obese participants (40%) and reduced consumption among non-obese participants by 10%.
	Capacci *et al.*^23^	Review of simulation studies	USA, Denmark, UK, France and Sweden	Aim: to provide a classification of public policies to promote healthy eating and mapping of existing measures in Europe Outcomes: saturated fat intake and cases of CHD and ischaemic strokes	Subsidies modelling (1): a 1% decrease in the price of all fruits and vegetables could translate into a mean decrease of around 6700 cases of CHD and almost 3000 ischaemic strokes. Saturated fat modelling (1): a tax of 14 DDK per kg of saturated fat using Danish data is estimated to reduce saturated fat intake by 7.4%. A tax on saturated fat would cause a small rise in salt intake as a result of cross-price elasticity effects and might overall result in more deaths than it averts. In addition, the saturated fat tax also results in a decrease in F/V consumption of ~2–4%
	Shultz *et al.*^24^	Systematic review	USA	Aim: to assess the effects of the 2009 food package revisions on healthy food and beverage availability, breastfeeding outcomes, and dietary intake of WIC programme participants 5 years later using only peer-reviewed research Outcomes: fruit intake	Significant changes were observed in dietary intake in another study, with increased fruit consumption by 0.33 servings per day among Hispanic mothers enrolled in WIC

**(b) Food promotion**					
Marketing/advertising to children	Gregori *et al.*^25^	Systematic review of RCTs	UK and USA	Aim: to perform a SR aimed at assessing the effect of TV advertising Outcomes: nutrition intake	4 RCTs: showed that total kcal intake was significantly higher and considerable more snacks were consumed after exposure to FA than after the NA. One RTC showed that the obese and overweight groups ate significantly more than the healthy weight group, both with FA and with NA
	Boyland and Halford^26^	Review	UK	Aim: to study the effect of TV advertising and branding on food preference and eating behaviour Outcomes: food intake	Two cross-sectional experimental studies found that exposure to food advertising increased food and calorie intake in all children. The obese and overweight groups ate significantly more than the healthy weight group, both with FA; and with NA
	Hingle and Kunkel^27^	Review	US focused	Aim: to analyze the role of food marketing and to evaluate intervention efforts to reduce exposure to advertising messages for unhealthy food products Outcomes: food consumption and obesity	The IOM report concluded that there is strong evidence that advertising influences the short-term food consumption of children aged 2–11 years; moderate evidence that it influences the regular diet of children aged 2–5 years; and weak evidence that it influences children aged 6–11 years. Economics study (1): estimates that the impact of banning food advertisements on television would reduce the baseline rate of childhood obesity in the USA from 17% to somewhere in the range of 10.5–14.5%
Nutrition education	Mytton *et al.*^28^	Systematic review and meta-analysis of RCTs	Europe, North America and India	Aim: to study the effects of either an increased or no increase in fruit and vegetable intake Outcomes: energy and fruit and vegetable intake	RCTs (8): No significant differences (*P*=0.93) were found in change in energy intake among studies supporting a general increase in F/V consumption (193 kJ) and studies providing a specific fruit portion per day (768 kJ). Two RCTs found that the difference in F/V intake between the arms (dietary advice vs no advice) were 294 g and 5.7 portions, with a follow-up duration of 4 and 52 weeks, respectively
	Harris *et al.*^29^	Systematic review of RCTs	USA, The Netherlands and Belgium	Aim: to assess the effectiveness of adaptive e-learning for improving dietary behaviours Outcomes: fruit and vegetable, total fat, saturated fat, total energy from fat, dietary fibre and daily energy intake	When studies reporting the same outcomes were pooled in a random effects meta-analysis, e-learning interventions were associated with a WMD of 0.24 servings (95% CI: 0.04 servings to 0.44 servings; *P*=0.019) of F/V per day(22 RCTs); WMD of –0.78 g (95% CI: –2.5 to 0.95 g) of total fat consumed (22 RCTs); WMD –0.24 g (95% CI: –1.44 to 0.96 g; *P*=0.7) of saturated fat intake (8 RCTs); WMD of –1.4% (95% CI: –2.5 to –0.3% *P*=0.012) of total energy consumed from fat; WMD of 4 kcal (95% CI: –85 to 93 kcal; *P*=0.93) of daily energy intake
	Thomson and Ravia^30^	Systematic review of clinical trials and RCTs	USA	Aim: to identify intervention trials designed to promote F/V intake Outcomes: fruit and vegetable intake	(RCTs): different nutrition education interventions were investigated and the effect on F/V. This review found increases in F/V intake that ranged from 0.4 to 1.4 servings per day
	Levy *et al.*^6^	Review	Mostly USA	Aim: to review the literature on school nutrition policies and price interventions Outcomes: SSB consumption	Two cross-sectional studies found no relationship to SSB consumption for nutrition education programs. RCT: SSB consumption over 3 days decreased by 0.6 servings in the intervention group receiving an ES curriculum compared with 0.2 servings in the control group. RCT: an education program aimed at discouraging SSB found that daily consumption of SSB decreased in the intervention group by ~20%, with almost no change in the control group
Campaigns	Rekhy and McConchie^31^	Review	Australia, USA, Denmark, UK and New Zealand	Aim: to examine the effect of campaigns and interventions Outcomes: fruit and vegetable intake	Australian *‘Go for 2&5'* campaign: analysis after 3 years showed that there was an average net increase of 0.8 serves per day (11.4%) for overall consumption of fruits (+0.2; 10%) and vegetables (+0.6; 12%). Modest impact on consumption behaviour in the long run. U.S. '5 A Day for Better Health' programme and *‘*'Fruits & Veggies – More Matters': survey results between 2004 and 2009 have shown that although F/V consumption for adults remained unchanged at 1.81 cups per person per day, it did increase for children <6 years old (7%) and between 6 and 12 years old (5%). Danish ‘6 a day' campaign: between ‘95 and ‘04, the survey reported that F/V consumption for the 4- to 10-year-old and 11- to 75-year-old group increased by 29%, 58%, 41% and 75%, respectively. For the period ‘03–‘08, the average intake of vegetables for adults (18–75 years old) was reported to be 162 g per person per day, whereas the average intake of fruit was 283 g per person per day. This equals 445 g per day, exceeding the minimum WHO requirements of 400 g per day, demonstrating the success of the Danish campaign U.K.'s ‘Food Dudes' programme: there is a 60–200% increase in F/V consumption and where monitored, an associated decline in consumption of unhealthy foods by 20–100%. New Zealand's ‘5+A Day' Programme: between '95 and '12 there was a reported increase of 23% in F/V intake of 5+ servings per day. In 2011, 60.4% of the population was reported to be consuming the recommended two serves of fruit daily and 66% of the population was consuming the recommended three serves of vegetables a day
	Snyder^32^	Review	Developing and developed countries	Aim: to review the evidence for the effectiveness of health communication campaigns to inform future nutrition campaign Outcomes: effect size, fruit and vegetable intake	Preliminary analysis of 37 F/V media campaigns found an average campaign effect size of *r*=0.08. Other SRs of F/V interventions found increases in F/V servings and decreasing fat per calories consumed. In-school nutritional campaigns aimed at fourth and fifth graders found an average effect of *r*=0.12
	Perez-Cueto *et al.*^33^	Review of policy documents	17 EU member states	Aim: to identify and assess healthy eating policies at national level Outcomes: food consumption and health	Nutrition education programs: the Portuguese PPC comprises training sessions for the adult population and reported reductions in total energy intake (−6.3%), cholesterol (−9.2%), total fat (−12.2%) and saturated fat (−15.6%) between baseline and follow-up. Public information campaigns: the Italian ‘Eat well, live healthy' campaign reported that 37.8% of the participants improved their dietary habits as a consequence of the campaign. The UK's ‘five-a-day' campaign showed that only 24% of surveyed individuals reported an increased F/V intake in the previous 6 weeks

**(c) Food provision**					
Workplace (fruit and vegetable and fat)	Mhurchu *et al.*^34^	Systematic review of RCTs	USA, Belgium and Holland	Aim: to assess the effect of worksite interventions Outcomes: intake	Fruit and vegetable intake: two RCTs showed that combined average daily intake of fruit and vegetable increased from +3 to +16% in intervention groups, compared with −2 to +4% in the control groups. Total fat intake: five RCTs showed that average daily intake of total fat as a percent of energy decreased from −2.2 to −9.1% in intervention groups compared with +1.3 to −1.8% in the control groups
	Montano *et al.*^35^	Review—meta-analysis of RCTs	Not mentioned	Aim: to assess the types of SEP in RCTs and estimate the moderation of SEP in workplace intervention Outcomes: fruit and vegetable intake	36 RCTs: daily consumption of fruit and vegetables increased significantly (SMD 0.12, 95% CI: 0.01–0.22). There were no statistical significant differences between occupational classes and fruit and vegetable intake (SMD 0.117, 95% CI: −0.049 to 0.282, EM 0.000, 95% CI: −0.230 to 0.231)
	Thompson and Ravia^30^	Systematic review of clinical trials and RCTs	USA	Aim: to identify intervention trials designed to promote F/V intake Outcomes: fruit and vegetable intake	RCTs: results for seven different work-based interventions reported that increase in daily F/V servings ranged from 0 to 1.52 servings per day and averaged 0.8 servings a day
Schools (SSBs)	Levy *et al.*^6^	Review	Mostly USA	Aim: to review the literature on school nutrition policies and price interventions Outcomes: SSB consumption	Eliminating access: was predicted to reduce SSB consumption by 4% (longitudinal study). Another cross-sectional study found that eliminating access would lead to 0.16 fewer servings per day. Removing SSBs from cafeteria vending machines led to a 35% decline in SSB consumption, but they were obtained from non-cafeteria vending machines and home (time series). Another time-series study also found an increase of SSB consumption at home
	Patel and Cabana^36^	Review	USA, Germany and UK	Aim: to summarise school-based interventions and policies Outcomes: beverage intake	Cross-sectional (1): replacing SSBs with water is estimated to result in an average reduction of 235 kcal per day among 2–19 years old. Two European studies associated school drinking water provision and promotion with an increase in student water consumption, but no change in student SSB intake
Schools (fruit and vegetables)	Evans *et al.*^37^	Systematic review and meta-analysis	UK, USA, The Netherlands, New Zealand, Norway, Canada and Denmark	Aim: to quantify the impact of school-based interventions on fruit and vegetable intake in children aged 5–12 years Outcomes: fruit and vegetable intake	Fruit and vegetables: the results of the meta-analyses indicated an improvement of 0.25 portions (95% CI: 0.06, 0.43 portions) of fruit and vegetable daily intake if fruit juice was excluded and an improvement of 0.32 portions (95% CI: 0.14, 0.50 portions) if fruit juice was included. Improvement was mainly due to increases in fruit consumption but not in vegetable consumption. The results of the meta-analyses for fruit (excluding juice) and vegetables separately indicated an improvement of 0.24 portions (95% CI: 0.05, 0.43 portions) and 0.07 portions (95% CI: 20.03, 0.16 portions), respectively
Schools (fat and fruit and vegetables)	Jaime and Lock^38^	Systematic review of RCTs, non-RCTs and cross-sectional studies	USA and Europe	Aim: to review the effectiveness of school food and nutrition policies worldwide Outcomes: fat and fruit and vegetable intake	Fat intake (3): all guideline interventions targeting fat intake led to significant decreases in total fat (range −2.0 to −10.9% of energy) and saturated fat intakes (range −0.9 to −5.2% of energy) (1 RCT and 2 non-randomised not controlled trials) Fruit and vegetable intake: two RCTs targeting F/V intake led to significant increase from +0.30 to +0.37 servings per day. Providing a free piece of fruit per d resulted in an increase in F/V intake between +0.38 to +0.44 servings per day (cluster RCT). Furthermore, intake increased 117 g per d for the intervention group vs 67 g per d for the control group (cross-sectional) Effect in fruit intake: increase across reception and year 1 pupils of 0.4 portion (95% CI: 0.2–0.5) and 0.6 portion (95% CI: 0.4–0.9) at 3 months, which fell to 0.2 portion (95% CI: 0.1–0.4) and 0.3 portion (95% CI: 0.1–0.6) at 7 months, respectively (non-randomised non controlled trial)
	Delgado- Noguera *et al.*^39^	Systematic review and meta-analysis of RCTs and CCTs	USA, UK, the Netherlands, Ireland and Italy	Aim: to assess the effectiveness of school interventions Outcomes: fruit and vegetable consumption	Price interventions: providing subsidised F/V in schools led to an increase in consumption of +0.59 servings per day. Another study established a subsidised fruit tuck shop for 1 year in intervention schools, but the consumption of F/V did not increase. A pooled analysis of these two RCTs found no significant differences between intervention and control group (SMD 0.02, 95% CI: −0.08, 0.12; *I*^2^=0%). A CCT investigated giving each student one piece of F/V daily for 2 years. With this intervention, fruit intake increased by +0.2 servings after 3 months, but the effect dropped back to +0.1 servings at 7 months, and returned to baseline in the second year. Computer-based interventions: a CCT found significant increases in F/V consumption in the intervention schools, whereas two RCTs also found increased consumption. Pooled analysis of the two RCTs did find significant differences between the intervention and control groups (SMD 0.33, 95% CI: 0.16, 0.50; *I*^2^=0%)
	Stables *et al.*^40^	Review of pre–post-test studies	USA	Aim: to profile the 5-day interventions Outcomes: fruit and vegetable intake	Pre-test–post-test: four of the seven projects reported significant changes in vegetable and fruit consumption, ranging from a 0.2 to 0.7 serving net change between treatment and control groups
	Perez-Cueto *et al.*^33^	Review of policy documents	17 EU member states	Aim: to identify and assess healthy eating policies at national level Outcomes: fruit and vegetable intake	School (1): fruit consumption as a morning snack among children at MS increased (6.5% after vs 3.6% before). Fruit consumption in general also increased among adolescents (17% after vs 4% before), whereas SSB consumption decreased (3.3% after vs 9.6% before). Workplace (1): the Danish ‘six-a-day' intervention provided a free fruit to their employees between 2001 and 2003 daily fruit consumption increased by 3.42 units on average
Schools (salt)	Weichselbaum and Buttriss^41^	Review	UK	Aim: to review current dietary and lifestyle habits of school-aged children in the UK and considers the impacts of these habits Outcomes: sodium intake	Limit range of unhealthy products and provide healthier options: a mixed methods study with a repeat cross-sectional design found that healthier school lunches not only improved nutrient intake at lunch time, but that these were also associated with improvements in the overall diet of children aged 4–7 years. Sodium intakes decreased significantly between 2003/2004 and 2008/2009, from 2.0 g to 1.85 g per day. In 11–12 year olds there was a significant decrease in intake of sodium from 2.59 to 2.15 g per day

**(d) Food composition**					
** **Reformulation (trans fat)	Downs *et al.*^42^	Systematic review of ITS, post-test, pre–post-test and case–control studies	Denmark and the Netherlands	Aim: to systematically review evidence for the effectiveness of policies aimed at reducing industrially produced TFAs in food Outcomes: consumption	Voluntary reformulation: of TFA levels in the Netherlands were associated with a 20% reduction in dietary intake. Pre–post-test (1): energy intake in the form of TFAs decreased from 1.0% before reformulation to 0.8% after. TFA intake from fats was the same. Denmark's trans fat ban (pre–post-test): TFA intake decreased from 4.5 g per day in 1976 to 1.5 g per day in 1995 and TFAs were virtually eliminated from food in 2005 after the ban
	Mozaffarian and Clarke^43^	Meta-analysis of RCTs and prospective cohort studies	North America and Europe	Aim: to evaluate the effect of reduced trans fatty acid by means of reformulation Outcomes: consumption and CHD	RCTs (13): for PHVO with 20% TFA, replacement with soybean, canola or high oleic sunflower oils would produce the largest (8.8–9.9%) CHD risk reductions. For PHVO with 35% TFA, risk reductions for replacement fats and oils ranged from 11.9 to 16.0%, with vegetable oils producing the largest decline. Predicted risk reductions were greatest for replacement of PHVO with 45% TFA, including risk reductions of 18.7 (soybean) and 19.8% (canola oil). Prospective cohort studies (4): for PHVO with 20% TFA, replacement with cottonseed, soybean or canola oils would produce the largest reductions (19.0–21.8%) in CHD risk. For PHVO with 35% TFA, risk reductions for replacement fats and oils ranged from 14.4 to 33.4%, with vegetable oils producing the largest decline. Predicted risk reductions were greatest for replacement of PHVO with 45% TFA, including risk reductions of 39.6 (soybean) and 38.6% (canola oil). In prospective cohort studies, each 2% energy replacement of TFAs with SFAs, MUFAs or PUFAs would lower CHD risk by 17% (95% CI: 7–25%), 21% (95% CI: 12–30%) or 24% (95% CI: 15–33%), respectively
** **Reformulation	Livingstone *et al.*^44^	Review	Focus on UK	Aim: to examine the effects of milk and dairy products that have been modified through alteration of the dairy cow's diet Outcomes: absolute CHD and stroke risk. Cardiovascular events	One double-blinded, randomised, cross-over, intervention study modified the fat content through feeding and found that the SFA content was reduced by 16.1% and the MUFA and PUFA content was increased by 9.9% and 7.5%, respectively. The reductions in LDL levels would be equivalent to a reduction in absolute risk of CHD and stroke of 7% and 5%, respectively. A SR and meta-analysis of RCTs suggested that replacement of 9.9% of dietary energy from SFA with PUFA resulted in an overall pooled risk reduction of 19% (relative risk 0.81; 95% CI: 0.70, 0.95; *P* ¼ 0.008)

**(e) Food labelling**					
Menu labelling	Sinclair *et al.*^45^	Systematic review and meta-analysis of experimental and quasi-experimental studies		Aim: to determine whether the provision of menu-based nutrition information affects the selection and consumption of calories in restaurants and other foodservice establishments Outcomes: consumption	Menu labelling with calories alone did not have the intended effect on decreasing calories selected and consumed compared with the control group (−31 kcal (*P*=0.35) and −13 kcal (*P*=0.61), respectively) (pooled mean difference). Conversely, when conditions that provided additional contextual or interpretive information were examined, the pooled mean difference in calories selected was 67 fewer calories (95% CI: −116.99 to −17.79; *P*=0.008). Contextual or interpretive interventions resulted in a pooled mean difference in calories consumed of 81 fewer calories (95% CI: −138.99 to −22.36; *P*=0.007)
	Swartz *et al.*^46^	Systematic review of experimental and quasi-experimental studies	USA	Aim: to evaluate the effect of calorie labelling on food choice Outcomes: consumption	2 non-blind RCTs: Overall, participants did not differ significantly in the number of calories they consumed by menu type (no label 739, calorie labels 805, no value pricing 761 calories) (no label 1459 vs label 1335 vs calorie+information 1256). However, combining the two label menus vs the no label menu showed that the labelled condition consumed fewer calories (label 1286 vs no label 1466)
Nutrition labelling	Campos *et al.*^47^	Systematic review of cross-sectional, experimental, natural experiment and longitudinal studies	USA, Europe, Canada, Trinidad, Australia, New Zealand, UK and Thailand	Aim: to review research on consumer use and understanding of nutrition labels, as well as the impact of labelling on dietary habits Outcomes: fat consumption	Observational studies: several studies have reported an association between label use and lower-fat consumption, and reported that people are more likely to eat healthier varieties of foods. Experimental studies: participants tended to consume greater amounts of reduced energy food in terms of weight, however total energy intake was significantly lower
	Downs *et al.*^42^	Systematic review	Canada and USA	Aim: to review evidence for the effectiveness of policies aimed at reducing industrially produced TFAs in food	2 pre–post-tests: after mandatory labelling led to 76% of foods meeting voluntary TFA limits, intake in the population still exceeded the WHO recommendation that <1% of dietary energy intake should come from consuming TFAs. Blood: mean TFA level decreased from 43.7μmol l^−1^ in 2000 to 19.4 μmol l^−1^ in 2009 (that is, 58% decrease)
	Finkelstein *et al.*^48^	Review of experimental studies	England, USA	Aim: to present and discuss seven proposed intervention strategies to promote healthy eating Outcomes: energy and fat intake	Nutrition labelling: a lab-based study providing nutrition information on labels during meals had no impact on energy intake. Similarly, a controlled experiment in a restaurant setting found that provision of nutrition information had no effect on overall energy and fat intake
	Hawley *et al.*^49^	Review	the Netherlands	Aim: to review the literature on front-of-pack labelling and supermarket shelf-labelling system Outcomes: consumption	Cross-sectional lab-based design: one study researched the effect of the choices logo on chocolate mousse cake to address the ‘health halo' concern and found that the cake was perceived as ‘less unhealthy', but there was no difference in taste perception or cake consumption compared with not having the logo. This suggests that the logo does not promote overconsumption.
	Vyth *et al.*^50^	Review of modelling studies	European countries, UK, Australia, New Zealand, USA, Canada	Aim: to evaluate the methodological quality of current front-of-pack labelling research and discuss future research challenges Outcomes: intake and health	Modelling studies: replacing typical daily menus and foods by choices menus and choices compliant foods can potentially lead to improved nutrient intakes towards recommendations

**(f) Food supply chain, trade and investment**					
Trade and market liberalisation	Friel *et al.*^51^	Review	Pacific Island countries, Central and North America	Aim: to review the available evidence on the links between trade agreements, food environments and diets from an obesity and NCD perspective Outcomes: soft drink consumption, NCD risk	Economic modelling study: demonstrated that liberalisation of FDI through trade agreements with the USA significantly increased the amount of soft drinks consumed within signatory LMICs, and consequently increased the risk of NCDs, particularly diabetes. Of key concern in the Pacific region has been the increasing import of fatty products and the, therefore, rising saturated fat intakes. For example, between 1963 and 2000, total fat supply increased by 80% in some Pacific Island countries
	Traill *et al.*^52^	Review	High, high–middle, middle–low and low-income countries	Aim: to describe and discuss the dietary and nutritional changes that have occurred since the 1992 International Conference on Nutrition by attempting to untangle the multitude of factors that have contributed to such changes	Overall, evidence indicates the price-induced impact of trade liberalisation on consumption and diets has been modest. The World Bank has estimated that even complete trade liberalisation would raise prices of agricultural commodities by only 5.5%, and, thus, such results as the URAA's achievement of only partial liberalisation are unlikely to have resulted in significant dietary change. The share of processed food in food and agricultural exports grew from 54 to 69% for high-income countries and from 49 to 67% for Asia between the 1970 s and 2000 s. Although models indicate relatively minor price changes for agricultural commodities, trade liberalisation may have induced other, less quantifiable changes in food supply systems. In India, market liberalisation in the mid-1990 s stimulated a rapid increase in imports of low-priced vegetable oils, which corresponded with a simultaneous increase in consumption
Monetary subsidies and taxes	Thow *et al.*^21^	Systematic review of empirical and modelling studies	USA, UK, Sweden, Denmark, Norway, Egypt, France, Ireland and Scotland	Aim: to assess the effect of food taxes and subsidies Outcomes: diet and health status	Modelling study (1): seven scenarios for taxing unhealthy and subsidising healthy foods and nutrients in Denmark were examinated. Each involved the equivalent of halving VAT on F/V. It was concluded that subsidising specific nutrients was more effective than subsidising food groups. Their best revenue-neutral scenario decreased average consumption of sugar by 6.5%, fat by 2.5% and saturated fat by 3.6%. Modelling study (1): it was predicted that increasing soft drink prices in Norway by 27% would reduce average consumption in heavy (44%) and light soft drink consumers (17%). One study modelled extending the VAT at 17.5% in the UK and Northern Ireland to food products that were the main sources of saturated fat. They calculated that saturated fat consumption would reduce deaths from ischaemic heart disease by 1.8–2.6%, or 1800–2500 deaths per year in the country
	Galizzi^9^	Review of modelling studies	USA and UK	Aim: to assess the relative effectiveness of taxes Outcomes: ischaemic risks	Modelling study (1): an increase in VAT up to 17.5% on fat foods in the USA can reduce ischaemic risks of 1.8–2.6% with more a 1000 lives saved a year
Climate change	Lake *et al.*^53^	Review	Developed countries (using the UK as a case study)	Aim: to investigate the potential impact of climate change on food security Outcomes: human health and obesity	The likely impact of climate change on world food prices: rises in food price associated with climate change may reduce the nutritional quality of dietary intakes because individuals may shift to lower-cost food items (less healthy choices) and can lower the nutritional status of some groups. It can also increase the risk of obesity, particularly among children, young adults, smokers, lower-income groups and frail older people.

**(g) Multi-component interventions**					
Food promotion and provision	Blanchette and Brug^54^	Review	USA, Norway, Denmark and UK	Aim: to review the current literature on interventions to increase the consumption of fruits and vegetables among children Outcomes: fruit and vegetable intake	The results of the interventions showed daily increases in F/V intake from 0 to 2.54 daily servings, with 14 of the 15 interventions resulting in increased consumption of fruit and/or vegetables. Increases in fruit intake were more frequent and substantial than increases in vegetable intake
	Patel and Cabana^36^	Review	USA, Germany and UK	Aim: to summarise school-based interventions and policies Outcomes: beverage intake	Replacing SSBs with water: two RCT studies associated school drinking water provision and promotion with an increase in water consumption, but no change in SSB intake
	Epstein *et al.*^12^	Review of experimental research	Not mentioned	Aim: to review experimental research on policy decisions Outcomes: dietary intake and health	Experimental study (1): A 20% subsidy on healthy food, together with educational materials and blood pressure readings led to a 6% increase in consumption of healthy foods and a 2% reduction of unhealthy foods. Consumption of healthy foods increased by 17% at follow-up with maintenance of the 2% decrease in unhealthy food consumption
Labelling and voluntary reformulation (trans fat)	Downs *et al.*^42^	Systematic review of ITS, post-test, pre–post-test and case–control studies	Canada	Aim: to review evidence for the effectiveness of policies aimed at reducing industrially produced TFAs in food Outcomes: dietary intake	Interrupted time series (1): mandatory TFA labelling combined with voluntary limits was associated with a 30% reduction in dietary intake in the general population. TFA intake: mean TFA intake decreased from 8.4 g per day in the mid-1990 s to 3.4 g per day in 2008, which is still above WHO recommendations. On average, there was a 30% decrease in TFA intake between 2004 and 2008. SFA intake did not increase during this period

Abbreviations: CBA, Controlled Before- and After studies; CCTs, Controlled Clinical Trials; CHD, Coronary Heart Disease; CVD, Cardiovascular Disease; ES, Elementary School; EU, European Union; FA, food advertisements; FDI, Foreign direct investment; IOM, Institute of Medicine; ITS, Interrupted Time Series; LDL, low-density lipoprotein; LMICs, Low and middle income countries; MS, Middle school; MUFA, monounsaturated fatty acids; NA, non-food advertisements; NCD, non-communicable disease; PE, Price Elasticity; PHVO, partially hydrogenated; PPC, Portuguese 'Peso Comunitario' vegetable oils; PUFA, polyunsaturated fatty acids; RCT, randomised controlled trials; SEP, Socioeconomic Positions; SFA, saturated fatty acids; SMD, Standardised Mean Difference; SR, systematic reviews; SSB, sugar-sweetened beverages; TFA, trans fatty acids; URAA: Uruguay Round Agreement on Agriculture; VAT, value-added tax; WHO, World Health Organisation; WMD, weighted mean difference.
