# Supplementary material for: The effects of policy actions to improve population dietary patterns and prevent diet-related non-communicable diseases: scoping review
Source: Eur J Clin Nutr. 2016 Nov 30;71(6):694–711. doi: 10.1038/ejcn.2016.234 (PMC5470099; doi:10.1038/ejcn.2016.234)
Supplement: Supplementary File 1 [file ejcn2016234x1.docx]

**Supplementary file 1. Data extraction tables.**

Table 1a. Food Price

*Taxes (SSBs)*

| **Study ID** | **Study Design** | **Study Aim** | **Policy(ies) Analysed** | **Participants & sample size** | **Geographical Scope** | **Methods** | **Outcomes** | **Study Limitations** | **Comments** |
| --- | --- | --- | --- | --- | --- | --- | --- | --- | --- |
| **Cabrera Escobar 2013**^55^ | A meta-analysis | To evaluate the literature on SSB taxes or price increases, and their potential impact on consumption levels, obesity, overweight and body mass index (BMI) | 1. Tax on SSBs |  | No restrictions | Databases were searched for papers that reported changes in diet or BMI, overweight and/or obesity due to a tax on, or price change of SSBs | 9 papers were included for meta-analysis  Own price elasticities Of the studies done in middle income countries, the one in Brazil showed an elasticity of −0.85 and the one in Mexico, -1.085. The results from the meta- analysis show that the pooled elasticity estimate is −1.30 (95% CI: -1.089 – -1.509)  Cross price elasticities For fruit juices the meta-analysis shows cross-price elasticity of 0.388 (95% CI: 0.010 – 0.767), for whole milk a cross-price elasticity of0.129 (95% CI: -0.085 – 0.342) and for diet soft drinks the cross-price elasticity is −0.423 (95% CI: -0.628 – -0.219). This means that fruit juices and perhaps whole milk act as substitutes for SSBs and diet soft drinks are consumed in complement to SSBs  A 1% increase in SSB price, the point prevalence for obesity would reduce more in men (−0.34 percentage points) than in women (−0.05) | In LMICs, consumption patterns and price sensitivities may differ from HIC (although the evidence from Mexico and Brazil does not support this)  The data included in the meta-analysis are from heterogeneous populations with various data sources involving households, individuals as well as a range of food stores and vending machine outlets | Only studies in English were included. This excludes potential useful studies conducted in different languages |
| **Study ID** | **Study Design** | **Study Aim** | **Policy(ies) Analysed** | **Participants & sample size** | **Geographical Scope** | **Methods** | **Outcomes** | **Study Limitations** | **Comments** |
| **Levy et al. (2011)**^22^ | Review of RCTs and population studies | To review the literature on school  nutrition policies and price interventions directed at youth SSB consumption | 1. Tax on SSBs |  | US, UK, Belgium, Brazil, Canada, Holland | Studies were collected using searches of PubMed, the Social Science Index, and Social Science Research Network by using various combinations of the terms BMI, overweight, obesity, SSB, school, nutrition policies/interventions, and price. In addition, references were identified from pertinent articles, including recent reviews | A 1 percentage point increase in the vending machine tax rate was associated with a 0.006-kg/m2 reduction in BMI among adolescents at risk of being overweight (P = 0.09)  One study examined the effect of taxes on young school children and found limited effects on soda consumption or BMI, although stronger effects were observed for those with high income and those with high BMI  Another study found no effect of soda taxes on BMI or the probability that a youth consumes soda, but a 1% increase in the tax rate was associated with nearly 8 fewer kcal from soda consumed (P < 0.05), an approximate 6% reduction  No differences were found in obesity rates between states with and without a >5% tax  One study found that a 10% increase in the price of sugar products was associated with a decrease in the prevalence of overweight by 2% and of obesity by 8%  *Demand studies*  A recent review concluded that the price elasticity for soft drinks is in the range of 20.8 to 21.0. For low-income consumers, a 10% price increase was associated with an 8% reduction in soda consumption, with little effect of soda prices on the consumption of other beverages  A 20% SSB price increase was found to reduce SSB energy intake by 38.8% for adults and 48.8% for youth, with an offset of 1.9% for adults and 6.1% for youth  Estimates from one study imply that a tax increase at 20% of current prices would reduce consumption by between 16 and 20%. A 16–20% reduction in youth SSB consumption would translate into a reduction of 36–45 kcal, assuming average intake of 225 kcal | It is not considered how the effects of a policy may depend on other policies or how the effects vary by socio-economic indicators |  |
| **Study ID** | **Study Design** | **Study Aim** | **Policy(ies) Analysed** | **Participants & sample size** | **Geographical Scope** | **Methods** | **Outcomes** | **Study Limitations** | **Comments** |
| **Welsh et al. (2013)**^10^ | Review article | To discuss current data on (SSB) consumption trends, evidence of the health impact, and the role of industry in efforts to reduce the consumption | Excise taxes |  | US |  | Replacement of one serving of SSBs and fruit juices per day with one cup of plain water was associated with 7 and 8% lower risk of type 2 diabetes  A recent study estimated the potential impact on health and spending of a nationwide penny-per-ounce excise tax on SSBs. It was estimated that the tax would reduce the consumption of SSBs by 15% among adults and prevent 2.4 million diabetes person-years, 95 000 coronary heart events, 8000 strokes, and 26 000 premature deaths, while avoiding more than $17 billion in medical costs  Beverage companies oppose taxes and pay community interest groups to lobby on their behalf |  | No methods section included in the paper |

*Taxes (Fat)*

| **Study ID** | **Study Design** | **Study Aim** | **Policy(ies) Analysed** | **Participants & sample size** | **Geographical Scope** | **Methods** | **Outcomes** | **Study Limitations** | **Comments** |
| --- | --- | --- | --- | --- | --- | --- | --- | --- | --- |
| **Cash Lacanilao (2007)** ^56^ | Review | To review the economic evidence of using taxes to achieve desirable health outcomes | 1. Taxes |  |  | Search and review the evidence on the ‘fat tax’ and highlight some of the theoretical and pragmatic issues around such interventions | The results of a possible fat tax show that an increase of 0.4-1% would not significantly affect consumption or health outcomes |  |  |
| **Study ID** | **Study Design** | **Study Aim** | **Policy(ies) Analysed** | **Participants & sample size** | **Geographical Scope** | **Methods** | **Outcomes** | **Study Limitations** | **Comments** |
| **Galizzi (2012)**^11^ | Review | To assess the relative effectiveness of each type of intervention | 1. Regulation and taxation |  |  |  | Few economic studies have simulated the likely effects of possible fat taxes. For instance, it has been calculated that, in the US, an increase in VAT up to 17.5% on fat foods can reduce ischemic risks of 1.8-2.6% with more a 1000 lives saved a year.  A simulation of the introduction of fat tax in the UK based on actual calories consumption, found that 2% of poorest consumers would pay 7 times as much the proportion of their income as the 2% richest consumers  It has also been calculated that a tax proportional to fat content can reduce fat intake of 1%, and that burden on poor consumers would be 10 times higher |  | No methods section |
| **Study ID** | **Study Design** | **Study Aim** | **Policy(ies) Analysed** | **Participants & sample size** | **Geographical Scope** | **Methods** | **Outcomes** | **Study Limitations** | **Comments** |
| **Tiffin & Salois (2012)**^12^ | Review | To assess the influence of socio-demographic factors on nutrition and health status and consider the impacts of nutrition policy across the population drawing on methodologies from both public health and welfare economics | 1. Fat tax |  |  |  | We investigate a policy that increases the price of fatty foods by 1% for every percent of saturated fats they contain; for instance, milk that contains 1.72% of saturated fats will see its price increasing by 1.72%  We put a ceiling on the price increase of 15%. To offset this tax burden and to encourage consumption of fruit and vegetables, a subsidy on fruit and vegetables is introduced, so as to exactly cancel the costs of the fat tax paid by consumers  We have analysed the impacts of a fiscal food policy on nutrient intakes. It is seen that firstly the policy induces only a small change in intakes and secondly that there is little difference in the changes across different social groups. The policy will therefore have limited impact on the inequalities that exist between different socio economic groups in terms of their nutrient intakes |  | No methods section |

*Taxes (fat & sugar)*

| **Study ID** | **Study Design** | **Study Aim** | **Policy(ies) Analysed** | **Participants & sample size** | **Geographical Scope** | **Methods** | **Outcomes** | **Study Limitations** | **Comments** |
| --- | --- | --- | --- | --- | --- | --- | --- | --- | --- |
| **Faulkner et al. (2011)**^57^ | Scoping review | To synthesize existing evidence regarding the impact of economic policies targeting obesity and its causal behaviors (diet, physical activity), and to make specific recommendations for the Canadian context | 1. Financial measures such as prices, taxes and subsidies |  | No restriction | Arksey and O’Malley’s (2005) methodological framework for conducting scoping reviews was adopted for this study and this consisted of two phases: 1) a structured literature search and review, and 2) consultation with experts in the research field through a Delphi survey and an in-person expert panel meeting in April 2010  Only observational or RCTs were included | 38 studies were identified  States without a soft drink or snack food tax were four times more likely (albeit statistically insignificant p-value = 0.25) than states with a tax to exhibit a high relative increase in obesity prevalence  A 10% increase in the price of fruits and vegetables was associated with a 0.7% increase in child BMI  Fruit and vegetable price elasticity for BMI was estimated to be 0.25 for the full sample and 0.60 among low-income children  An increase in the price of fruits and vegetables by one standard deviation raised children’s BMI by 0.11 units by third grade (equivalent to a BMI price elasticity of approximately 0.05) | Generalisation was limited due to the use of the delphi method  The BMI used was self-reported which can lead to bias  Furthermore the price data used in this study is also subject to bias due to data collection is higher income areas so hard to match to low and middle income areas |  |

*Tax (high energy dense foods)*

| **Study ID** | **Study Design** | **Study Aim** | **Policy(ies) Analysed** | **Participants & sample size** | **Geographical Scope** | **Methods** | **Outcomes** | **Study Limitations** | **Comments** |
| --- | --- | --- | --- | --- | --- | --- | --- | --- | --- |
| **Epstein et al. (2012)**^45^ | Review – experimental research | To review experimental research that can provide evidence base for policy decisions that influence food purchasing, dietary intake, and health. | 1. Fiscal measures; taxes, subsidies, price elasticity |  |  | Literature searches were conducted to identify original research articles that examined food and/or nonalcoholic beverage purchases as a function of price manipulation in at least one phase of their design | 24 studies met the inclusion criteria  Results showed that a 50% price increase in a lab setting resulted in a reduction in calories of 16%  20% subsidy on healthy food, together with educational materials and blood pressure readings led to a 6% increase in consumption of healthy foods and a 2% reduction of unhealthy foods | Experimental research can have unintended effects | Most studies looked at the effect of fiscal measures on purchases (which is not an outcome for our review) |

*Tax (sugar)*

| **Study ID** | **Study Design** | **Study Aim** | **Policy(ies) Analysed** | **Participants & sample size** | **Geographical Scope** | **Methods** | **Outcomes** | **Study Limitations** | **Comments** |
| --- | --- | --- | --- | --- | --- | --- | --- | --- | --- |
| **Powell &** **Chaloupka (2009)**^58^ | Review | To assess the relationship between food prices (or taxes or subsidies) and weight outcomes (BMI) | 1. Price elasticities, taxes and subsidies |  | US | Literature was searched for quantitative empirical studies to investigate the relationship between fiscal measures and weight outcomes using US data | One study showed that a one-dollar increase in the current price of sugar was associated with a 0.20 and 0.33 percentage point reduction in the probability of overweight and obesity, respectively  Based on the summary statistics provided in the paper, the marginal effects correspond to a current price of sugar elasticity of −0.20 for overweight and −0.81 for obesity  An increase in the price of fruits and vegetables by one standard deviation raised their BMI by 0.11 units by third grade (equivalent to a BMI price elasticity of approximately 0.05) | Price data used is limited as it is collected in cities (not always the same ones) and uses higher income areas. This can pose a problem when comparing to low and middle income areas  Food survey – only a small number of food products are surveyed  Data used in a number of studies was based on self-reporting which may have biased the results towards the null |  |

*Subsidies (fruit & vegetables)*

| **Study ID** | **Study Design** | **Study Aim** | **Policy(ies) Analysed** | **Participants & sample size** | **Geographical Scope** | **Methods** | **Outcomes** | **Study Limitations** | **Comments** |
| --- | --- | --- | --- | --- | --- | --- | --- | --- | --- |
| **Black et al. (2012)**^59^ | Systematic review | To summarise the evidence for the health and nutrition impacts of food subsidy programmes among disadvantaged families from high income countries | 1. Food subsidies | Socio-economically disadvantaged adults, children or families living independently in the community in high income countries | USA, UK and New Zealand | Search for RCTs, CBAs and ITS studies. The Cochrane handbook was followed | In the community center F&V RCT (n=40), one study reported that overweight women who received US$10/week of F&V for six months increased mean consumption of fruit by 1.0 serves/day (95% CI 0.1-1.9, p=0.02) and vegetables by 0.9 serves/ day (95% CI 0.3-1.5, p=0.002) compared to controls, whose intake was unchanged  Another study assessed the impact of free home-delivered fruit juice and/or nutrition education throughout prenatal care on the overall fruit intake of pregnant females in a RCT (n=190) in the UK. Among those who received subsidized juice, there was a net increase of 34.8% in the proportion consuming more fruit juice, while the intake of fruit juice declined among the control (−7.9%) | Risk of bias as there were 10 non-randomized studies  Most studies reported multiple nutritional outcomes without specifying a primary outcome, which increases the likelihood of significant associations occurring by chance  Most studies used self-reporting as a method which can lead to recall bias and imprecision  No data on men |  |
| **Study ID** | **Study Design** | **Study Aim** | **Policy(ies) Analysed** | **Participants & sample size** | **Geographical Scope** | **Methods** | **Outcomes** | **Study Limitations** | **Comments** |
| **An (2012)**^60^ | Review article | To systematically review evidence from field interventions on the effectiveness of monetary subsidies in promoting healthier food purchases and consumption | 1. Monetary subsidies | Adolescents 12–17 years old or adults 18 years and  older | USA, Canada, France, Germany, Netherlands, South Africa and the UK | Search for literature with the following criteria: intervention: field experiments; (ii) randomized controlled trials, cohort studies or pre–post studies;(iii) subsidy: price discounts or vouchers for healthier foods; (iv) outcome: food purchases or consumption; (vi) period: 1990–2012; and (vii) language: English  Quality assessed using predefined methodological criteria | 24 papers on 20 distinct experiments were included  There is some preliminary evidence from price discount interventions that the demands for fruits and low-fat snacks are price elastic – a 1 % decrease in price is associated with a larger than 1 % increase in quantity demanded  A 10 % and 25 % discounts on healthier food purchases were associated with an increase in daily fruit/vegetable intake by 0.38 and 0.64 servings, respectively | Only studies in English are included  The external validity of study outcomes. Almost all studies were limited in scale, had a small or convenience sample rather than a population-representative sample, and were implemented in very specific settings, which have substantially limited the generalization of the study results beyond the sample  The duration of the interventions were limited and no follow up period, so long term effects cannot be evaluated  Impact on energy intake limited as diet quality was not assessed | Most data was on sales numbers rather than dietary intake |

*Price elasticities*

| **Study ID** | **Study Design** | **Study Aim** | **Policy(ies) Analysed** | **Participants & sample size** | **Geographical Scope** | **Methods** | **Outcomes** | **Study Limitations** | **Comments** |
| --- | --- | --- | --- | --- | --- | --- | --- | --- | --- |
| **Andreyeva et al. (2010)**^7^ | Systematic review | To assess mean elasticities by food category and variations in estimates by study design | Food prices – price elasticity |  | United States | Searching the literature for US-based studies on the price elasticity of demand for major food categories to determine mean price elasticities by category and assess variations in estimates by study design | 160 studies were included in this review, of which 99 studies used time series data, 34 studies used household survey data and 27 studies used scanner data  All mean price elasticity estimates from the food categories were below 1.0 and ranged from 0.27 to 0.81. The higher the number, the more responsive to changes in price and more elastic  Soft drinks were considered most elastic, followed by juice, meats, fruit, cereals, eggs, sugar and sweets, cheese and fats and oils. Some categories had a smaller number of data points, which means that the findings must be interpreted with caution | No separate estimates of price elasticity for fruit and vegetables as they were combined, which may have led to underestimation of the separate price elasticities for the demand for fruit and vegetables  As there was a lack of elasticity estimate standard errors, no meta-analysis could be conducted and therefore a simplified calculation of means was used |  |
| **Study ID** | **Study Design** | **Study Aim** | **Policy(ies) Analysed** | **Participants & sample size** | **Geographical Scope** | **Methods** | **Outcomes** | **Study Limitations** | **Comments** |
| **Maniadakis et al. (2013)**^61^ | Systematic Review | To assess the possible effects of such taxation policies by identifying and analyzing all studies which investigate the impact of price increases on consumption, caloric intake and weight outcomes | 1. Taxation of foods |  | No restriction | Literature was searched for original studies including any of the four types of primary research – existing data, experiments, surveys and observations – with the focus on the association between SSBs and HFSSFs prices and taxes and their corresponding consumption or energy intake or obesity related outcomes | 55 studies were included in this review, of which 22 demand studies, 11 longitudinal studies, 11 cross-sectional studies, 6 modelling studies, 4 experimental studies and 1 cohort studies  **Demand studies** The price elasticity of demand for beverages is in the range of -0.5 to -1.6, depending on the beverage considered, with most of them falling below 1.0  The caloric effect of a 10% increase in prices or a corresponding imposition of a tax reduces energy intake by a maximum of 50 calories per day, 450 per month, and up to 0.3 kilograms or 1.5 pounds per year, which cannot be considered significant  **Longitudinal studies** The price elasticity of demand for beverages and foods is in the range of -0.05 to -0.35 depending on the beverage and food considered | Transformation of consumption figures to energy and weight outcomes was often based on extrapolation models, which require careful consideration  Total energy intake was an outcome measure, which is partial to substitution effects  Populations may not have been representative and different studies have been undertaken in different settings |  |
| **Study ID** | **Study Design** | **Study Aim** | **Policy(ies) Analysed** | **Participants & sample size** | **Geographical Scope** | **Methods** | **Outcomes** | **Study Limitations** | **Comments** |
| **Powell et al. (2013)**^15^ | Systematic review | To assess the price elasticity of demand for sugar-sweetened beverages (SSBs), fast food and fruits and vegetables as well as the direct associations of prices/taxes with body weight outcomes | Price elasticities |  | US | Search the literature for studies to identify the price elasticity of demand for SSBs. | The review identified 14 studies that estimated price effects for SSB and soft drink demand with 10 studies that provided price elasticity of demand measures  The mean SSB price elasticity estimate of −1.21 implies that a tax that raises the price of SSBs by 20% would reduce overall consumption of SSBs by 24%  The mean price elasticity of demand for regular carbonated soda of −1.25 (range −0.71 to −2.26)27;29–31 suggested that a 20% increase in price would reduce consumption by 25%  The mean price elasticity for fruit and vegetables was −0.49 and −0.48, respectively, suggesting that subsidizing fruits and vegetables by 20% would increase consumption by 10% |  | This paper is not taking substitute effects into account |

*Taxes and subsidies*

| **Study ID** | **Study Design** | **Study Aim** | **Policy(ies) Analysed** | **Participants & sample size** | **Geographical Scope** | **Methods** | **Outcomes** | **Study Limitations** | **Comments** |
| --- | --- | --- | --- | --- | --- | --- | --- | --- | --- |
| **Eyles et al. (2012)**^13^ | Systematic review of simulation studies | To investigate the estimated association between food pricing strategies and changes in food purchases or intakes (consumption) ; Health and disease outcomes and whether there are any differences in these outcomes by socio-economic group | 1. Food pricing strategies |  | The 34 member countries of the OECD | Simulation modelling studies were searched to map the relationship between food price change and resultant change in at least one of the following components: food consumption, health status and NCDs | The estimated mean own-PE, which represents the change in demand with a 1% change in price, for carbonated soft drinks was -0.93 (range, -0.06, -2.43)  For the three studies assessing a flat rate tax, the mean modelled reduction in saturated fat consumption in response to a 1% increase in price was -0.02% (range, -0.01%, -0.04%) energy from saturated fat  Overall findings of fruit and vegetable subsidies were pro-health and the estimated mean own-PE was -0.35 (range, -0.21, -0.77). A 0.35% increase in fruit and vegetable purchases is expected for each 1% reduction in price  After sensitivity analysis the own PE is -0.54 (range -0.23, -0.77) | Only 22% of the studies included were of moderately high quality  The majority of the studies (19) did not include a cross price elasticity which may have affected the results  Only papers in English were included and member countries of the OECD |  |
| **Study ID** | **Study Design** | **Study Aim** | **Policy(ies) Analysed** | **Participants & sample size** | **Geographical Scope** | **Methods** | **Outcomes** | **Study Limitations** | **Comments** |
| **Mozaffarian et al. (2012)**^14^ | Systematic review and grading of evidence | Summarize evidence for effective population approaches |  |  |  |  |  |  |  |
|  |  |  | US taxes on sugars and syrups | 17 states | US |  | No studies on outcome |  |  |
|  |  |  | SR on price and demand/consumption | 160 studies | US | SR | Strong association on food away from home, soft drink, juice and meats |  |  |
|  |  |  | Tax reviews |  |  | Modelling and real-world | Little effect with low tax, large effect with higher tax |  |  |
|  |  |  | Subsidy |  |  | Cross-sectional, modelling and interventional | Partially offset by substitution to other foodstuffs  10% reduction in price might cause 2-5% increase consumption (US) |  |  |
|  |  |  |  |  |  | Observational and prospective | Tax/Subsidies might prevent 6000 CHD/cancer deaths per year (UK)  Price more effective than promotion and may be sustainable (after subsidy ends) |  |  |
|  |  |  | Price generally |  |  | Small-scale interventions | Decreased CHD in Finland with decreased animal fat price. Similar in former Soviet countries |  |  |
| **Study ID** | **Study Design** | **Study Aim** | **Policy(ies) Analysed** | **Participants & sample size** | **Geographical Scope** | **Methods** | **Outcomes** | **Study Limitations** | **Comments** |
| **Thow et al. (2010)**^16^ | Systematic review | To assess the effect of food taxes and subsidies on diet, body weight and health through a systematic review of the literature | Monetary subsidies or taxes levied on specific food products | Not available | No restriction | Searching the English and grey literature for empirical studies and modelling studies investigating the effect of a tax or subsidy on food consumption, body weight or disease  Empirical studies were defined as assessing the effect of an actual tax, while modelling studies predicted the outcomes of potential taxes or subsidies | 24 studies were included in this review, of which 16 were modelling studies and 8 empirical studies. The effect of taxes on food consumption alone was assessed by 9 studies. 5 studies assessed the effect on consumption and body weight, and 6 on body weight only  Overall, consumption, body weight and disease incidence were altered by taxes and subsidies in the way it was expected, with higher taxes being associated with greater effects  Some studies focused on only one dietary component rather than the entire diet, which did not consider substitute effects. Therefore the effect on consumption and body weight may have been overestimated | The review’s findings are limited by the high proportion of modelling studies, which are based on assumptions and subject to data limitations  Only English language studies were included and majority of evidence came from high-income countries |  |
| **Study ID** | **Study Design** | **Study Aim** | **Policy(ies) Analysed** | **Participants & sample size** | **Geographical Scope** | **Methods** | **Outcomes** | **Study Limitations** | **Comments** |
| **Thow et al. (2014)**^8^ | Systematic review | To assess the effect of food taxes and subsidies on consumption | Taxes and subsidies |  | High income countries | Databases were searched for literature that focused on taxes and subsidies and assessed the effect on consumption | *Subsidies on healthy foods*  One RCT conducted in supermarkets in New Zealand found that a subsidy of 12.5% increased healthy food purchases by around 10%, with little to no effect on unhealthy nutrient consumption  Similarly, a study of stated preferences from the United States found a subsidy of 50% on fruit and vegetables would increase consumption by 25%  *Subsidies; modelling*  Four studies that modelled fruit and vegetable subsidies of around 10% showed increases in consumption of around 5%, with one study estimating a 1.5% increase in consumption in response to a 1.8% price decrease | Only English papers were included  The lack of studies from low- and middle-income countries  The wide variety of targets of taxation that have been proposed and modelled add uncertainty to the conclusions that can be drawn regarding public health and policy measures  This study is also limited by its focus on assessments of fiscal policy interventions, which means that other, possibly relevant studies that focused only on price would have been excluded |  |
| **Study ID** | **Study Design** | **Study Aim** | **Policy(ies) Analysed** | **Participants & sample size** | **Geographical Scope** | **Methods** | **Outcomes** | **Study Limitations** | **Comments** |
| **Capacci et al. (2012)**^9^ | Structured review | To provide a classification of public policies to promote healthier eating as well as structured mapping of existing measures in Europe | 1. Fiscal measures: taxes and subsidies |  | Europe | Policy reviews, grey literature, policy documents and websites were searched for interventions at macro level and healthy eating (defined as adherence to the nutrition recommendations of the WHO14 and the maintenance of normal weight) | A 1% decrease in the price of all fruits and vegetables could translate into a mean decrease of around 6,700 cases of coronary heart disease and almost 3,000 ischemic strokes  **Simulation effects**  Using Danish data, a 7.4% reduction in saturated fats is estimated in response to a tax of 14 DKK per kilogram of sat fat  A 10% increase in prices of dairy products reduces total fat intake from dairy products by 0.86% only, while the total revenue from the tax would be about $ 4.5 billion  A tax on saturated fat would cause a small rise in salt intake as a result of cross-price elasticities of demand and might overall result in more deaths than it averts. It also would cause a decrease in fruit and vegetable consumption of approximately 2–4% as a result of cross-elasticity effects | The US was not included in this study but they seem to be further on certain policies, such as (state-level) fiscal measures | This review only looked at policies across Europe and some countries have more advanced policies than others |
| **Study ID** | **Study Design** | **Study Aim** | **Policy(ies) Analysed** | **Participants & sample size** | **Geographical Scope** | **Methods** | **Outcomes** | **Study Limitations** | **Comments** |
| **Schultz et al. (2015)^65^** | Systematic review | To assess the effects of the 2009 food  package revisions on healthy food and beverage availability,  breastfeeding outcomes, and dietary intake of WIC program  participants 5 years later using only peer-reviewed research | Vouchers (subsidies) |  | US | Four electronic databases were searched between April 1 and 30, 2014, for peer-reviewed research. Two reviewers screened the articles, extracted the data, and established inter-rater reliability by discussing and resolving discrepancies. | Significant changes were observed in dietary intake in another study, with increased fruit consumption by 0.33 servings per day among Hispanic mothers enrolled in WIC | The limited search  terms used in the literature review may not have  retrieved all articles relevant to the 2009 WIC food  package revisions  Relatively few studies were abstracted for their review |  |

Table 1b. Food Promotion

*Marketing/advertising to children*

| **Study ID** | **Study Design** | **Study Aim** | **Policy(ies) Analysed** | **Participants & sample size** | **Geographical Scope** | **Methods** | **Outcomes** | **Study Limitations** | **Comments** |
| --- | --- | --- | --- | --- | --- | --- | --- | --- | --- |
| **Gregori et al. (2014)**^17^ | Systematic review of RCTs | To perform a SR of evidence coming from RCTs aimed at assessing the effect of television advertising on food intake in children from 4 to 12 years old | TV advertising |  |  | Randomized controlled trials were searched in PubMed database and included if they assessed the effect of direct exposure to television food advertising over the actual energy intake of children | 7 studies were included in the review | Bias results, limiting the possibility to generalise to population level  Small samples and very specific age groups | Standardized methods are needed |
|  |  |  |  | 66 children aged 5–7 years old | UK | Children watched a 14-min cartoon embedded with a selection of each type of advert. After watching, they were offered a snack consisting of pre-weighed individual servings of six snack foods on a plate | For all children, total kcal intake was higher following the unhealthy food ads compared to both the healthy food ads and toy ads |  |  |
|  |  |  |  | 59 children aged 9-11 years old | UK | Children were exposed to food or non-food ads and presented with plates of food afterwards, able to eat as much or little as they wanted | Total kcal intake was significantly higher after exposure to food advertising compared to the control group. A significant interaction with BMI status and advertisement type was observed |  |  |
|  |  |  |  | 42 children aged 9-11 years old | UK | Children watched a collection of food or non-food ads followed by a 10min cartoon. They were asked which ads they had seen and presented with four food categories, able to eat as much or as little as they wanted | The obese and overweight groups ate significantly more than the healthy-weight group, both with food ads (FA) and with non-food ads (NA) |  |  |
|  |  |  |  | 118 children aged 7-11 years old | US | Children watched a 14min episode including either food or non-food ads. They received snacks while watching | The group exposed to  cartoon and ads ate  considerably more  snacks, compared to the  group exposed to  non-food advertisement |  |  |
|  |  |  |  | 93 children aged 5-7 years old | UK | Children were exposed to a collection of 10 non-food or food ads, followed by a 10-min cartoon. After viewing, each child was presented with pre-weighed individual servings of five food categories | Total kcal intake was significantly higher after exposure to food advertisements (FA) than after the non-food advertisements (NA). |  |  |
|  |  | To study the role of maternal pressure to control weight gain as a potential modulator of the children’s eating as a reaction to food advertising. |  | 121 children aged 8-12 years old | Netherlands | Living room setting; children watched a 20min movie clip interrupted by different ads and they could freely eat from the bowl with food provided | The study showed that kids, who were encouraged from their mothers to be thinner, ate more especially when exposed to light food commercials than when exposed to neutral advertising. In contrast, children who perceived no maternal pressure ate more when exposed to neutral commercials than when exposed to either energy-dense or light food commercials |  |  |
|  |  |  |  | 118 children aged 7-11 years old | US | The children watched a 14-min episode of a cartoon, included commercials: one-half were randomized to the “food commercial condition” and one-half to the “non-food commercial condition”. Children received a pre-weighed bowl of snacks to consume while watching | The group exposed to  cartoon and ads ate  considerably more  snacks, compared to the  group exposed to  non-food advertisement |  |  |
| **Study ID** | **Study Design** | **Study Aim** | **Policy(ies) Analysed** | **Participants & sample size** | **Geographical Scope** | **Methods** | **Outcomes** | **Study Limitations** | **Comments** |
| **Boyland & Halford (2013)**^18^ | Review | To study the effect of TV advertising and branding on food preference and eating behaviour in children | Food advertising to children | Children |  |  | Food advertising has often been proposed as a candidate for the association between television viewing and adiposity. In support of this, another study found a significant, positive correlation between levels of overweight in nine countries (seven EU countries, the US and Australia) and the number of television adverts for sweet or fatty foods broadcast in a 20 h period  A study showed that there was a significant association between commercial viewing in 1997 and BMI z-score in 2002 for children aged 0–6 years  A study found that exposure to food advertising increased food intake in all children. The obese and overweight groups ate significantly more than the healthy-weight group, both with food ads (FA),x2 (2) = 24.3; p < 0.001; and with non-food ads (NA), x2 (2) = 22.0; p< 0.001 |  | No methods section present and no regular clear structure |
| **Study ID** | **Study Design** | **Study Aim** | **Policy(ies) Analysed** | **Participants & sample size** | **Geographical Scope** | **Methods** | **Outcomes** | **Study Limitations** | **Comments** |
| **Hingle & Kunkel (2012)**^19^ | Review | To analyse the role of food marketing as a contributor to childhood obesity and young people’s screen time and weight status. They also evaluate intervention efforts to reduce exposure to advertising messages for unhealthy food products | Food advertising to children | Children |  |  | The most comprehensive review of studies examining the effects of food marketing on children was conducted by the IOM in 2006. The IOM report concluded that there is strong evidence that advertising influences the short-term food consumption of children aged 2 to 11 years, there is moderate evidence that advertising influences the regular diet of children 2 to 5 years of age, and weak evidence that it influences children 6 to 11 years of age; and there is strong evidence that exposure to advertising is associated with adiposity in people aged 2 to 18  *Economics study*  Estimates that the impact of banning food advertisements on television would reduce the baseline rate of childhood obesity in the United States from 17% to somewhere in the range of 10.5% to 14.5% |  | Methods are not clearly reported |

*Nutrition education*

| **Study ID** | **Study Design** | **Study Aim** | **Policy(ies) Analysed** | **Participants & sample size** | **Geographical Scope** | **Methods** | **Outcomes** | **Study Limitations** | **Comments** |
| --- | --- | --- | --- | --- | --- | --- | --- | --- | --- |
| **Mytton et al. (2014)**^21^ | Systematic review and meta-analysis | To study the effect of an increased  vegetable and fruit intake on either body weight or  energy intake observed in randomised trials in free-  living human populations compared to no increase in  vegetable and fruit intake | Dietary advice | 1026 adults | Europe, North America and India | Searched for only RCTs to include in the analysis | 8 studies were included  Two types of studies were investigated; type a: those that encouraged or supported a general increase in fruit and vegetable consumption vs type b: those that provided a specific fruit portion to be consumed on a daily basis  For the outcome of change in energy intake, there was a smaller increase in energy intake among type a studies (change in energy intake: 193 kJ, 95% CI: −284 to 672) than for type b studies (change in energy intake: 768 kJ, 95% CI: 274 to 1263), although the differences were not significant (p = 0.93)  2 studies investigated the effect of dietary advice on increasing fruit and vegetable intake. The daily difference in fruit and vegetable intake between the arms were 294g and 5.7 portions, with a follow up duration of 4 and 52 weeks respectively | All studies relied wholly or largely on standard questionnaires or re-call to record diet intake (including energy intake) rather than objective measures  None of the studies were double blind  Study duration typically short |  |
| **Study ID** | **Study Design** | **Study Aim** | **Policy(ies) Analysed** | **Participants & sample size** | **Geographical Scope** | **Methods** | **Outcomes** | **Study Limitations** | **Comments** |
| **Harris et al. (2011)**^23^ | Systematic review of RCTs | To assess the effectiveness and cost-effectiveness of adaptive e-learning interventions for dietary behaviour change, and also to explore potential psychological mechanisms of action and components of effective interventions | Nutrition education – e-learning |  | US, Netherlands and Belgium | Electronic bibliographic databases were searched for the period January 1990 to November 2009. Reference lists of included studies and previous reviews were also screened; authors were contacted and trial registers were searched. Studies were included if they were randomized controlled trials, involving participants aged ≥ 13 years, which evaluated the effectiveness of interactive software programs for improving dietary behaviour | 43 studies were included in this systematic review  When studies reporting the same outcomes were pooled in a random effects meta-analysis, e-learning interventions were associated with a Weighted Mean Difference (WMD) of +0.24 (95% CI 0.04 to 0.44) servings of fruit and vegetables per day; -0.78 g (95% CI -2.5 g to 0.95 g) total fat consumed per day; -0.24 g (95% CI -1.44 g to 0.96 g) saturated fat intake per day; -1.4% (95% CI -2.5% to -0.3%) of total energy consumed from fat per day; +1.45 g (95% CI -0.02 g to 2.92 g) dietary fiber per day; +4 kcal (95% CI -85 kcal to 93 kcal) daily energy intake; -0.1 kg/m2 (95% CI -0.7 kg/m2 to 0.4 kg/m2) change in body mass index | All studies took place in high income countries which limits the generalizability to other middle and low income countries |  |
| **Study ID** | **Study Design** | **Study Aim** | **Policy(ies) Analysed** | **Participants & sample size** | **Geographical Scope** | **Methods** | **Outcomes** | **Study Limitations** | **Comments** |
| **Thomson & Ravia (2011)**^20^ | Systematic review | To identify behaviour-based intervention trials designed to promote F/V intake | Behavior based interventions (nutrition education) |  | US | Literature was searched for interventions focusing on fruit and vegetables | Different nutrition education interventions were investigated and the effect on fruit and vegetables. This review found increases in F/V intake that ranged from 0.4-1.4 servings a day | Only papers in English were included, RCTs and clinical trials  Studies used self-reported fruit and vegetable intake  The issue of cost, an identified barrier to greater intake, was not addressed in these studies |  |
| **Study ID** | **Study Design** | **Study Aim** | **Policy(ies) Analysed** | **Participants & sample size** | **Geographical Scope** | **Methods** | **Outcomes** | **Study Limitations** | **Comments** |
| **Levy et al. (2011)**^22^ | Review of RCTs and population studies | To review the literature on school  nutrition policies and price interventions directed at youth SSB consumption | 1. School nutrition policies |  | Mostly US | Studies were collected using searches of PubMed, the Social Science Index, and Social Science Research Network by using various combinations of the terms BMI, overweight, obesity, SSB, school, nutrition policies/interventions, and price. In addition, references were identified from pertinent articles, including recent reviews | No relationship was found between SSB consumption and nutrition education programs in one study. Another study found that having a school policy against SSB access was associated with 18% less SSB consumption but found no relationship with education programs in either primary or secondary schools  *Randomized controlled trial*  SSB consumption over 3 d decreased by 0.6 servings (0.8 oz. or 236 mL) in the intervention group receiving an ES curriculum while increasing by 0.2 servings in the control group  For an education program aimed at discouraging SSB among Brazilian students aged 9–12 y, they found that daily consumption of carbonated SSB decreased in the intervention group by ~20%, with almost no change in the control group |  |  |

*Campaigns*

| **Study ID** | **Study Design** | **Study Aim** | **Policy(ies) Analysed** | **Participants & sample size** | **Geographical Scope** | **Methods** | **Outcomes** | **Study Limitations** | **Comments** |
| --- | --- | --- | --- | --- | --- | --- | --- | --- | --- |
| **Rekhy & McConchie (2014)**^24^ | Review | To examine the effect of campaigns and interventions aimed at fruit and vegetable intake | Campaigns |  |  |  |  | Self-reported data  Comparison difficult due to different measures, variables and outcomes |  |
|  |  |  |  | Western Australian adult  population | Australia | Health Department’s Health & Well-  being Surveillance System Surveys | *Australian “Go for 2&5” campaign*  Analysis of the campaign after 3 years showed that there was an average net increase of 0.8 serves per day for overall consumption of fruits and vegetables or 11.4% increase of the total recommended intake of fruits and vegetables. This included a 0.2 serve increase for fruit (10% of recommended fruit intake) and 0.6 serve increase for vegetables (12% of recommended vegetable intake) during this period, demonstrating only a modest impact on the consumption behaviour over the long term among the target group |  |  |
|  |  |  |  | Mothers of children aged  10 years and below | US | Consumption data collected every  5 years from The National Panel of  Diaries (NPD) – Nutrient Intake  Database | *U.S. “5 A Day for Better Health” programme and “Fruits & Veggies – More Matters”*  Survey results between the period 2004 and 2009 have shown that although fruit and vegetable consumption for individuals remained unchanged at 1.81 cups per person per day, it did increase for children less than 6 years old and between 6 and 12 years old, by 7% and 5% respectively  Fruit and vegetable consumption for adult males has declined by 2% (females 1%) between 2004 and 2009; fruit and vegetable consumption for males 18–34 years old and 35–44 years old and females 18-44 years old has increased by 4%; 2% and 4% respectively between 2004 and 2009  Fruit and vegetable consumption for 65 + years old has declined by 7–9% between 2004 and 2009 |  |  |
|  |  |  |  | Groups that ate the least fruit and vegetables –  families with children; men;  and young | Denmark | Data from Danish National Survey of  Dietary Habits and Physical Activity | *Danish “6 a day” campaign*  Between 1995 and 2004, the Danish National Survey of Dietary Habits and Physical Activity reported that vegetable and fruit consumption for the 4- to 10-year-old group increased by 29% and 58% respectively  For the 11- to 75-year-old group, vegetable and fruit consumption increased by 41% and 75% respectively, during the same period  For the period 2003–2008, the average intake of vegetables for adults  (18–75 years old) was reported to be 162 g per person per day, while the average intake of fruit for this group stood at 283 g per person per day  This equals 445g per day, exceeding the minimum WHO requirements of 400g per day, demonstrating the success of the Danish campaign |  |  |
|  |  |  |  | 2- to 11-year-olds (primary school children) | UK, Italy and US | Controlled trials and ongoing evaluations after the initial  intervention, in Primary Schools and  Nurseries/Early Years Centres | *U.K.’s “Food Dudes” programme*  There is a 60–200% increase in fruit and vegetable consumption and where monitored, an associated decline in consumption of unhealthy foods by 20–100%. The impact is highest among children who are the poorest eaters with significant success in “Special Schools |  |  |
|  |  |  |  | Household shoppers and  children | New Zealand | Telephone surveys, face-to-face street intercept, online surveys, Nielsen data | *New Zealand’s “5 + A Day” Programme*  In 1995, 31% of the population reported consuming 5+ servings a day.  In 2012, 38% reported consuming 5+ servings a day, an increase of 23% over 1995.  In 2011, 60.4% of the population was reported to be consuming the recommended two serves of fruit daily (a rise from 46% in 1997) and 66% of the population was consuming the recommended three serves of vegetables a day (no significant change since 1997) |  |  |
| **Study ID** | **Study Design** | **Study Aim** | **Policy(ies) Analysed** | **Participants & sample size** | **Geographical Scope** | **Methods** | **Outcomes** | **Study Limitations** | **Comments** |
| **Snyder (2007)**^25^ | Review | To review the evidence for the effectiveness of health communication campaigns  to inform future nutrition campaign | Campaigns |  | Global | This review draws on meta-analysis and systematic reviews that examine the effectiveness of communication campaigns and the scientific literature on health campaigns | Preliminary analysis of 37 fruit and vegetable media  campaigns found an average campaign effect size of r =.08 Other systematic reviews of fruit and vegetable interventions found increases in fruit and vegetable servings and decreasing fat per calories consumed. In-school nutritional campaigns aimed at fourth and fifth graders found an average effect of r = .12 |  | Limited display of results related to the effectiveness of nutrition campaigns |
| **Study ID** | **Study Design** | **Study Aim** | **Policy(ies) Analysed** | **Participants & sample size** | **Geographical Scope** | **Methods** | **Outcomes** | **Study Limitations** | **Comments** |
| **Perez-Cueto et al. (2012)**^26^ | Review of policy documents | To identify and assess healthy eating policies at national level which have been evaluated in terms of their impact on awareness of healthy eating, food consumption, health outcome or cost/benefit | Nutrition education and public information campaigns |  | Europe (17 EU member states) | Data were collected systematically in seventeen out of the twenty-seven EU Member States. The information on the policy interventions was gathered from governmental websites, through general search in databases and peer-reviewed journals, by personal contacts with national public servants and in direct consultation with policy makers. In addition, previous reviews and EU-funded projects were also consulted | 107 (out of 121) policies were retained  *Nutrition education programmes:* the Portuguese PPC comprises training sessions for the adult population and reported reductions in total energy intake (-6.3%), cholesterol (-9.2%), total fat (-12.2%) and saturated fat (-15.6%) between baseline and follow-up  *Public information campaigns:* the Italian ‘Eat well, live healthy’ campaign reported that 37.8% of the participants improved their dietary habits as a consequence of the campaign  The UK’s ‘five-a-day’ campaign showed that only 24% of surveyed individuals reported an increased F/V intake in the previous six weeks |  |  |

Table 1c. Food Provision

*Workplace (Fruit & vegetable and fat)*

| **Study ID** | **Study Design** | **Study Aim** | **Policy(ies) Analysed** | **Participants & sample size** | **Geographical Scope** | **Methods** | **Outcomes** | **Study Limitations** | **Comments** |
| --- | --- | --- | --- | --- | --- | --- | --- | --- | --- |
| **Mhurchu et al. (2010)**^31^ | Systematic review | To assess the effects of worksite interventions on employee diets | Workplace interventions |  |  | Searching databases for literature on workplace interventions and the effect on dietary outcomes | 16 studies (10 RCTs; 1 quasi-experimental with non-randomized design; 5 uncontrolled interventions – pre-test post-test design) were included in this review with follow up durations varying between 12 weeks to 2.5 years. Studies were targeted at employee education, environmental changes or both | Restricted to English papers and the use of limited databases  Study methodological quality was moderate  Self-reported data and sales data was used |  |
|  |  |  |  |  |  | RCT | *Fruit and vegetable intake*  Two RCTs that measured proportional change in combined fruit and vegetable intakes, average daily increases ranged from +3% to +16% in intervention groups, compared to -2% to +4% in control groups  *Total fat intake*  Five RCTs that measured proportional change in total fat as a percent of energy by intervention group, average daily reductions ranged from -2.2% to -9.1% in intervention groups compared with +1.3% to -1.8% in control groups |  |  |
| **Study ID** | **Study Design** | **Study Aim** | **Policy(ies) Analysed** | **Participants & sample size** | **Geographical Scope** | **Methods** | **Outcomes** | **Study Limitations** | **Comments** |
| **Montano et al. (2014)**^30^ | Review – meta analysis | To assess what types of socio-economic positions (SEP) are being considered in randomized controlled intervention studies and estimate the moderation of SEP in workplace intervention  effects on body mass index (BMI), fruit and vegetable consumption, musculoskeletal symptoms, and job stress | Worksite interventions |  |  | RCTs were identified to allow calculations of SMD. Workplace interventions investigating the effect on BMI, fruit and vegetable intake, musculoskeletal symptoms and job stress were included | 36 studies were included in this review. In total 40 reports of intervention effects were considered  Daily consumption of fruit and vegetables increased significantly (SMD 0.12, 95% CI 0.01–0.22)  There were no statistical significant differences between occupational classes and fruit and vegetable intake (SMD 0.117, 95% CI -0.049–0.282, EM 0.000, 95% CI -0.230–0.231) | Relevant studies may have been  omitted as a consequence of the keywords selected in the search strategy  The sample description in terms of occupational  class was very heterogeneous, thus limiting the validity  of the applied EPG classification |  |
| **Study ID** | **Study Design** | **Study Aim** | **Policy(ies) Analysed** | **Participants & sample size** | **Geographical Scope** | **Methods** | **Outcomes** | **Study Limitations** | **Comments** |
| **Thomson & Ravia (2011)**^20^ | Systematic review | To identify behaviour-based intervention trials designed to promote F/V intake | Behavior based interventions (nutrition education) |  | US | Literature was searched for interventions focusing on fruit and vegetables | Different nutrition education interventions were investigated and the effect on fruit and vegetables. This review found increases in F/V intake that ranged from 0.4-1.4 servings a day | Only papers in English were included, RCTs and clinical trials  Studies used self-reported fruit and vegetable intake  The issue of cost, an identified barrier to greater intake, was not addressed in these studies |  |

*Schools (SSBs)*

| **Study ID** | **Study Design** | **Study Aim** | **Policy(ies) Analysed** | **Participants & sample size** | **Geographical Scope** | **Methods** | **Outcomes** | **Study Limitations** | **Comments** |
| --- | --- | --- | --- | --- | --- | --- | --- | --- | --- |
| **Levy et al. (2011)**^22^ | Review of RCTs and population studies | To review the literature on school nutrition policies and price interventions directed at youth SSB consumption | 1. Eliminating access to SSBs in schools |  | Mostly US | Studies were collected using searches of PubMed, the Social Science Index, and Social Science Research Network by using various combinations of the terms BMI, overweight, obesity, SSB, school, nutrition policies/interventions, and price. In addition, references were identified from pertinent articles, including recent reviews | For 5th graders, one study found that, of the 40% of children who had access, 26% consumed SSB at schools. Having access in schools increased the odds of consuming any SSB in or out of school in the previous week by 40%. As a result, eliminating access was predicted to reduce the percent who consumed SSB by 4%, with greater reductions among Black non-Hispanics (6%) and girls (5%)  For California, adolescents in schools without SSB in vending machines consumed 0.16 fewer servings/d  Upon removing LNED from snack bars and in cafeteria vending machines, student reported mean SSB school intake declined 35%, but slightly more SSB were obtained from non-cafeteria vending machines and home |  |  |
| **Study ID** | **Study Design** | **Study Aim** | **Policy(ies) Analysed** | **Participants & sample size** | **Geographical Scope** | **Methods** | **Outcomes** | **Study Limitations** | **Comments** |
| **Patel & Cabana (2010)**^27^ | Review | To describe the beverages currently offered within child care facilities and schools; and summarizes school and child care based interventions and policies to encourage healthy beverage intake | Healthy beverages in school and child care settings |  | US |  | A slightly different approach for preventing obesity via beverage consumption is to encourage water intake among children  In a National Health and Nutrition Examination Survey (NHANES) study, it was estimated that replacing all SSBs with water could result in an average reduction of 235kcal per day among 2-19 year olds  Two European studies associated school drinking water provision and promotion with an increase in student water consumption, but no change in student SSB intake |  | No clear methods section/ structure |

*Schools (fruit & vegetables)*

| **Study ID** | **Study Design** | **Study Aim** | **Policy(ies) Analysed** | **Participants & sample size** | **Geographical Scope** | **Methods** | **Outcomes** | **Study Limitations** | **Comments** |
| --- | --- | --- | --- | --- | --- | --- | --- | --- | --- |
| **Evans et al. (2012)**^29^ | Systematic review and meta-analysis | To quantify the impact of school-based interventions on fruit and vegetable intake in children aged 5-12 y | School based interventions | 26,361 children aged 5–12 y | UK, US, Netherlands, New Zealand, Norway, Canada, Denmark | A systematic literature review was carried out to identify randomized and nonrandomized controlled trials that were based in primary schools and designed to increase portions of daily fruit and vegetable intake. MEDLINE, Cochrane libraries, EMBASE, PsycINFO, and Educational Information Centre were searched from 1985 to 2009. Data were extracted, and mean effect sizes were calculated by using random effects models | The results of the meta-analyses indicated an improvement of 0.25 portions (95% CI: 0.06, 0.43 portions) of fruit and vegetable daily intake if fruit juice was excluded and an improvement of 0.32 portions (95% CI: 0.14, 0.50 portions) if fruit juice was included  Improvement was mainly due to increases in fruit consumption but not in vegetable consumption. The results of the meta-analyses for fruit (excluding juice) and vegetables separately indicated an improvement, of 0.24 portions (95% CI: 0.05, 0.43 portions) and 0.07 portions (95% CI: 20.03, 0.16 portions), respectively | Many studies published in this area are of poor-quality design without a control group or with poor randomization methods that lead to biased reporting  The reporting of results was not consistent, and a number of studies did not report both fruit and vegetable consumption combined  Successful programs may not have been included in the analysis because of a lack of suitable published data on improvements in fruit and vegetable intake over the whole day  There was some evidence of publication bias |  |

*Schools (fat and fruit & vegetables)*

| **Study ID** | **Study Design** | **Study Aim** | **Policy(ies) Analysed** | **Participants & sample size** | **Geographical Scope** | **Methods** | **Outcomes** | **Study Limitations** | **Comments** |
| --- | --- | --- | --- | --- | --- | --- | --- | --- | --- |
| **Jaime & Lock (2009)**^28^ | Systematic review of RCTs, non-RCTs and cross-sectional studies | To review the effectiveness of school food and nutrition policies worldwide in improving school food environment, student’s dietary intake and decreasing overweight and obesity | School interventions aimed at fat and fruit and vegetable intake |  | US and Europe | A literature search was conducted to identify (un)published studies of food or nutrition policies in preschools and school settings  Cluster RCT  Cross-sectional | 18 studies were included in this review  *Fat intake*  All guideline interventions targeting fat intake led to significant decreases in total fat (net effect ranged from -2.0% to -10.9% of energy) and saturated fat intakes (net effect ranged from -0.9% to -5.2% of energy)  *Fruit and vegetable intake*  Interventions targeting fruit and vegetable intake led to significant increase from +0.30 servings/day to +0.37 servings/day  **Price interventions**  Several studies looked at the effect of providing a free piece of fruit a day in schools. This resulted in an increase in fruit and vegetable intake between +0.38 to +0.44 servings a day. Compared to a control group, intake increased 117g/d for the intervention group vs 67g/d for the control group | Interventions conducted at a small no of schools  Interventions conducted developed countries |  |
| **Study ID** | **Study Design** | **Study Aim** | **Policy(ies) Analysed** | **Participants & sample size** | **Geographical Scope** | **Methods** | **Outcomes** | **Study Limitations** | **Comments** |
| **Delgado- Noguera et al. (2011)**^43^ | Systematic review and meta-analysis | To assess the effectiveness of school interventions for promoting the consumption of fruit and vegetables | School based interventions focused on fruit and vegetable intake |  | US, UK, Netherlands, Ireland and Italy | Search databases for RCTs and CCTs promoting fruit and vegetable intake in primary school setting | **Price interventions**  One study provided subsidized FV in the intervention schools. At the end of the second year, this intervention showed an increase in consumption of +0.59 servings of FV per day  Another study established a subsidized fruit tuck shop for one year in the intervention schools, but the consumption of FV did not increase. A pooled analysis of these two studies (1536 participants) found no significant differences between intervention and control groups (SMD 0.02, 95% CI − 0.08, 0.12; I²=0%)  A CCT investigated giving each student one piece of FV daily for two years. With this intervention, fruit intake increased by +0.2 servings after  three months, but the effect dropped back to +0.1 servings at seven months, and returned to baseline in the second year | Developed countries  Studies included are of moderate quality  Insufficient sample size  Selection bias in CCTs |  |
| **Study ID** | **Study Design** | **Study Aim** | **Policy(ies) Analysed** | **Participants & sample size** | **Geographical Scope** | **Methods** | **Outcomes** | **Study Limitations** | **Comments** |
| **Stables et al. (2005)**^44^ | Review | To profile the 5 a day interventions | 5 a day school based interventions | 7-14 years of age, ranging from 164 to 2600 participants |  | Grants were awarded to implement and evaluate the 5 a day programme. Based on the final report of 14 studies they were assessed for inclusion. Components mostly include education  The included studies were evaluated using a quasi-experimental pre/post-test research design | 7 studies included in this review  Four of the seven projects reported significant changes  in vegetable and fruit consumption, ranging from a 0.2- to 0.7-serving net change between treatment and control groups | Children and adolescent diets – hard to generalise validity and reliability  Self-reported data is used  Lacked rigor details regarding methodology and measurements |  |
| **Study ID** | **Study Design** | **Study Aim** | **Policy(ies) Analysed** | **Participants & sample size** | **Geographical Scope** | **Methods** | **Outcomes** | **Study Limitations** | **Comments** |
| **Perez-Cueto et al. (2011)**^26^ | Review of policy documents | To identify and assess healthy eating policies at national level which have been evaluated in terms of their impact on awareness of healthy eating, food consumption, health outcome or cost/benefit | School and workplace interventions |  | Europe (17 EU member states) | Data were collected systematically in seventeen out of the twenty-seven EU Member States. The information on the policy interventions was gathered from governmental websites, through general search in databases and peer-reviewed journals, by personal contacts with national public servants and in direct consultation with policy makers. In addition, previous reviews and EU-funded projects were also consulted | 107 (out of 121) policies were retained  *School based intervention*  After the intervention, fruit consumption as a snack in the morning among children at the middle school increased (6.5% after v. 3.6% before); fruit consumption in general  also increased among adolescents (17% after  v. 4% before), while sweet beverages consumption decreased (3.3% after v. 9.6% before)  *Workplace intervention*  An example of workplace action is the Danish ‘six-a-day’ that consisted of an agreement with companies to provide free fruit to their employees. A behavioural change attributable to this campaign is the 700% increase in companies providing their employees with free fruit between 2001 and 2003(a total of 4986 workplaces, 10% being public) and a resulting daily fruit consumption of 3.42 units on average |  |  |

*Schools (salt)*

| **Study ID** | **Study Design** | **Study Aim** | **Policy(ies) Analysed** | **Participants & sample size** | **Geographical Scope** | **Methods** | **Outcomes** | **Study Limitations** | **Comments** |
| --- | --- | --- | --- | --- | --- | --- | --- | --- | --- |
| **Weichselbaum & Buttriss (2014)**^62^ | Review | To review current dietary and lifestyle habits of school-aged children in the UK and considers the impacts of these habits on short- and long-term health. It updates the BNF briefing paper | Food provision in schools | Children and adolescents | UK |  | *Limit range of unhealthy products and provide healthier options*  A study commissioned by the Public Health Research Consortium (England) found that healthier school lunches not only improved nutrient intake at lunch time, but that these were also associated with improvements in the overall diet of schoolchildren aged 4–7 years. Sodium intakes decreased significantly between 2003/2004 and 2008/2009, from 2.0 g sodium/day to 1.85 g/day  In 11–12 year-olds there was a significant decrease in intake of sodium from 2.59 to  2.15 g/day |  | No clear structure. Methods and conclusion/discussion not present |

Table 1d. Food Composition

*Reformulation (trans-fat)*

| **Study ID** | **Study Design** | **Study Aim** | **Policy(ies) Analysed** | **Participants & sample size** | **Geographical Scope** | **Methods** | **Outcomes** | **Study Limitations** | **Comments** |
| --- | --- | --- | --- | --- | --- | --- | --- | --- | --- |
| **Downs et al. (2013)**^32^ | Systematic review | To systematically review evidence for the effectiveness of policies, including self-regulation, aimed at reducing industrially  produced trans fatty acids (TFAs) in food | Voluntary reformulation & trans-fat bans |  | Brazil, Canada,  Costa Rica, Denmark, the Netherlands,  the Republic of Korea and the United  States of America | A systematic literature search was conducted using the Medline, Embase and Cinahl databases to identify peer-reviewed articles that examined the effect of a TFA policy | 26 studies were included in the review  Voluntary self-regulation of TFA levels in the Netherlands was associated with a 20% reduction in dietary intake  Denmark’s trans-fat ban  TFA intake decreased from 4.5g per day in 1976 to 1.5  g per day in 1995 and TFAs were virtually eliminated from food in 2005 after the ban  Energy intake in the form of TFAs decreased from 1.0% before reformulation to 0.8%  after; the contribution of task force foods to TFA consumption declined from 45% to 29%; the  decrease in TFAs mainly occurred in pastries, cakes, biscuits and snacks; TFA intake from fats and margarines was unchanged | All but three studies included in this review were conducted in high-income countries  Some studies in this review were limited because they used compliance with regulations or the TFA level in blood or breast milk as an intermediate out-come and did not examine TFA intake and its associated disease risk  Studies examining TFA policy interventions in a “real-world” setting may overestimate the resulting reduction in TFA levels because of sampling limitations  The studies in the review were not directly comparable | Focus is more on TFA levels in food products after reformulation than the effect on dietary intake or health outcomes |
| **Study ID** | **Study Design** | **Study Aim** | **Policy(ies) Analysed** | **Participants & sample size** | **Geographical Scope** | **Methods** | **Outcomes** | **Study Limitations** | **Comments** |
| **Mozaffarian & Clarke (2009)**^33^ | Meta – analysis of randomized controlled trials and of prospective cohort studies | To evaluate the effect of reduced trans fatty acid by means of reformulation and thereby consumption on coronary heart disease | Reformulation of products containing fatty acids | 518 participants | Unlimited | The effects on CHD risk was calculated replacing three different PHVO formulations (containing 20, 35 or 45% TFA) with other specific fats or oils likely to be used for replacement on the basis of the content of TFAs, SFAs, MUFAs and PUFAs in each fat and oil. Estimates were based on iso-caloric replacement of 7.5% of energy from PHVO in an individual’s diet | *Randomized controlled trials:*  For PHVO with 20% TFA, replacement with butter would result in a very small net decrease (2.7%) in CHD risk; while replacement with palm oil or lard would more modestly (7.6 and 6.0%) decrease risk. Conversely, replacement with soybean, canola or high oleic sunflower oils would produce the largest (8.8–9.9%) CHD risk reductions. For PHVO with 35% TFA, risk reductions for replacement fats and oils ranged from 11.9 to 16.0%, with the largest predicted declines in CHD risk for replacement with vegetable oils. Predicted risk reductions were greatest for replacement of PHVO with 45% TFA, including risk reductions of 18.7 and 19.8% for replacement with soybean and canola oil, respectively | Estimates were based on iso-caloric replacement of 7.5% of energy from  PHVO, but PHVO consumption may be higher or lower in  different populations  Calculations considered n3 and n6 PUFAs as having similar effects and SFAs were also considered together. It is possible that SFAs of different chain lengths may have different effects on cardiovascular risk | When comparing the results from the RCTs to the observational studies, the outcomes from the prospective cohort studies are higher that the RCTs |
|  |  |  |  | 4965 CHD cases prospectively ascertained among 139 836 participants | North America and Europe |  | *Prospective cohort studies:*  For PHVO with 20% TFA, replacement with butter would have little net effect on CHD risk (0.5% higher risk), while replacement with palm oil or lard would modestly (9.1 and 7.3%, respectively) decrease risk. Replacement with high oleic sunflower oil would reduce risk by 15.9%, and replacement with cottonseed, soybean or canola oils would produce the largest reductions (19.0–21.8%) in CHD risk. For PHVO with 35% TFA, risk reductions for replacement fats and oils ranged from 14.4 to 33.4%, with the largest predicted declines in CHD risk for replacement with vegetable oils. Predicted risk reductions were greatest for replacement of PHVO with 45% TFA, including risk reductions of 39.6 and 38.6% for replacement with soybean and canola oil, respectively | The calculations based on cohort studies are subject  to residual con-founding from other lifestyle factors and to measurement error in assessment of dietary consumption from questionnaires |  |

*Reformulation*

| **Study ID** | **Study Design** | **Study Aim** | **Policy(ies) Analysed** | **Participants & sample size** | **Geographical Scope** | **Methods** | **Outcomes** | **Study Limitations** | **Comments** |
| --- | --- | --- | --- | --- | --- | --- | --- | --- | --- |
| **Livingstone et al. (2012)**^40^ | Review | To examine the effects of milk and dairy products that have been modified through alteration of the dairy cow’s diet | Reformulation of SFA in milk and dairy products |  | Focus on UK | The present review will summarise the data from the human intervention studies that have used milk and dairy products with modified FA composition achieved through alteration of the diet given to the cows | Nine studies were included in this review  One double-blinded, randomised, cross-over, intervention study modified the fat content through feeding and found that the SFA content was reduced by 16·1 % and the MUFA and PUFA content was increased by 9·9 % and 7·5 %, respectively. The reductions in LDL levels would be equivalent to a reduction in absolute risk of CHD and stroke of 7 and 5 %, respectively  A SR and meta-analysis of RCTs suggested that replacement of 9·9 % of dietary energy from SFA with PUFA resulted in an overall pooled risk reduction of 19 % (relative risk 0·81; 95 % CI 0·70, 0·95; P ¼ 0·008). |  |  |

Table 1e. Food Labelling

*Menu labelling*

| **Study ID** | **Study Design** | **Study Aim** | **Policy(ies) Analysed** | **Participants & sample size** | **Geographical Scope** | **Methods** | **Outcomes** | **Study Limitations** | **Comments** |
| --- | --- | --- | --- | --- | --- | --- | --- | --- | --- |
| **Sinclair et al. (2014)**^35^ | Systematic review and meta-analysis of experimental and quasi experimental studies | To determine whether the provision of menu-based nutrition information affects  the selection and consumption of calories in restaurants and other foodservice establishments | Menu labelling |  |  | Databases were searched to identify all controlled experimental and quasi-experimental studies that reported the effect of informative, contextual, or interpretive menu labeling on calories selected or consumed. | Menu labelling with calories alone did not have the intended effect on decreasing calories selected and consumed compared to the control group (-31 kcal [P=0.35] and -13kcal [P=0.61], respectively) (pooled mean difference)  Conversely, when conditions that provided additional contextual or interpretive information were examined, the pooled mean difference in calories selected was 67 fewer calories (95% CI -116.99 to -17.79; P=0.008). Contextual or interpretive interventions resulted in a pooled mean difference in calories consumed of 81 fewer calories (95% CI -138.99 to -22.36; P=0.007). | The majority of the studies had methodological weaknesses  All studies measured immediate effect rather than long-term effect  The quasi-experimental designs were susceptible to confounding bias  The experimental studies also tended to recruit from a narrow segment of the population; most participants were young and relatively well educated  Some chance of publication bias |  |
| **Study ID** | **Study Design** | **Study Aim** | **Policy(ies) Analysed** | **Participants & sample size** | **Geographical Scope** | **Methods** | **Outcomes** | **Study Limitations** | **Comments** |
| **Swartz et al. (2011)**^34^ | Systematic review | To evaluate the effect of calorie labelling on food choice | Calorie menu labelling |  | No restrictions but limited to English-language papers | Literature was searched (after August 2006) for studies that looked at the effect of calorie menu labelling in restaurants or cafeteria’s on food purchase and consumption | *Laboratory settings*  One study found that participants overall did not differ significantly in the number of calories they consumed by menu type (no label 739, calorie labels 805, no value pricing 761 calories, p = 0.25). Subgroup analysis did demonstrate a difference in calories consumed. Men in groups with menus listing calorie information and those without value pricing consumed more calories than those with control menus (p = 0.01)  Another study also found no significant difference between calorie consumption when they examined consumption by menu type overall (no label 1459 vs. calorie label 1335 vs. calorie + information 1256, p = 0.12). However, when they combined the two calorie label menus and compared them to the no label menu, they did find those in the labeled condition consumed fewer calories than those in the no label condition (label 1286 vs.no label 1466, p=0.04) | Each study in this review had methodological limitations (e.g. unable to generalise and confounding factors)  Two of the included studies were judged to be of good quality, and five of were judged to be of fair quality | Update of previously conducted systematic review |

*Nutrition labelling*

| **Study ID** | **Study Design** | **Study Aim** | **Policy(ies) Analysed** | **Participants & sample size** | **Geographical Scope** | **Methods** | **Outcomes** | **Study Limitations** | **Comments** |
| --- | --- | --- | --- | --- | --- | --- | --- | --- | --- |
| **Campos et al. (2011)**^36^ | Systematic review | To review research on consumer use and understanding of nutrition labels, as well as the impact of labelling on dietary habits | Nutrition labelling |  | US, Europe, Canada, Australia, New-Zealand, UK, Netherlands, France, Germany, Thailand and Trinidad | The present review was restricted to studies that examined consumer behaviour related to nutrition labels on pre-packaged foods, published in English in peer-reviewed journals or research reports completed on behalf of government agencies. | 120 papers were included  Among the general population, use of labels is typically above 50%. Women, young people, higher income, Caucasians are associated with higher use of labels  *Observational studies*  Several studies have reported an association between label use and lower fat consumption. Label users are also more likely to eat healthier varieties of foods, and to have reduced Na, cholesterol and energy intakes, coupled with increased fibre ,Fe and vitamin C intakes | Relevant articles may not be included due to the rapidly growing evidence base  The articles included were disproportionally from high income Western countries and from North America  Important differences in labelling regulations were not fully examined |  |
|  |  |  |  |  |  |  | *Longitudinal studies*  A study found that BMI of nutrition label users fell significantly following implementation of the Act, with the greatest change among those with the highest BMI score | Hard to compare studies due to the large variety of study designs, measures and samples  Most of the data is based on self-reported data |  |
|  |  |  |  |  |  |  | A study comparing nationally representative surveys of consumers in 1989 and 1995 found that frequent label users in 1995 had a significantly greater probability of consuming a low-fat diet than both non-label users in 1995 and frequent label users in 1989. In addition, fat intake among less-educated respondents decreased significantly during the ‘pre–post’ study period |  |  |
|  |  |  |  |  |  |  | *Experimental studies*  Two experiments compared consumption of low-fat with energy-dense foods by randomizing participants to either a blind or information condition. Both studies found that, although participants tended to consume greater amounts of reduced-energy food in terms of food weight, total energy intake was significantly lower among those who consumed reduced-energy food  Participants who received nutrition information consumed more of the low-energy version of the food |  |  |
| **Study ID** | **Study Design** | **Study Aim** | **Policy(ies) Analysed** | **Participants & sample size** | **Geographical Scope** | **Methods** | **Outcomes** | **Study Limitations** | **Comments** |
| **Downs et al. (2013)**^32^ | Systematic review | To review evidence for the effectiveness of policies, including self-regulation, aimed at reducing industrially  produced trans fatty acids (TFAs) in food | Food labelling |  | No restrictions | Literature was searched to identify papers that examined the effect of a TFA policy. Only empirical studies conducted in a ‘real world’ setting were included | 26 studies were included of which 8 focused on labelling alone and 4 on labelling and voluntary limits  In Canada, mandatory  TFA labelling combined with voluntary limits was associated with a 30% reduction in dietary intake in the general population  In the US, mandatory TFA labelling was associated with a 58% reduction in TFA levels in blood plasma  In Canada, even after mandatory labelling led to 76% of foods meeting voluntary  TFA limits, intake in the population still exceeded the WHO recommendation  that less than 1% of dietary energy intake should come from consuming TFAs | Some studies in this review were limited because they used compliance with regulations or the TFA level in blood or breast milk as an intermediate outcome and did not examine TFA intake and its associated disease risk  Another limitation is that studies examining TFA policy interventions in a “real-world” setting may overestimate the resulting reduction in TFA levels because of sampling limitations  The studies in the review were not directly comparable | Majority of the outcomes were related to fatty acid composition rather than consumption or health outcomes  The paper gives as one of the strongest arguments for favouring mandatory TFA labelling because it will lead to product reformulation which is the focus |
| **Study ID** | **Study Design** | **Study Aim** | **Policy(ies) Analysed** | **Participants & sample size** | **Geographical Scope** | **Methods** | **Outcomes** | **Study Limitations** | **Comments** |
| **Finkelstein et al. (2004)**^63^ | Review | To present and discuss the relative merits of seven proposed interventions strategies to promote healthy eating | Nutrition labelling in restaurants and food away from home sources |  |  |  | A recent laboratory study of food intake among normal-weight women found that explaining the concept of energy density and providing nutrition information on labels during meals had no impact on energy intake  Similarly, a controlled experiment in a restaurant setting in England found that provision of nutrition information had no effect on overall energy and fat intake  Although consumers may have full knowledge of the health content of their FAFH meals, because they may lack information of how the health content is translated into actual health, consumers may still not make optimal food choices |  | No methods section |
| **Study ID** | **Study Design** | **Study Aim** | **Policy(ies) Analysed** | **Participants & sample size** | **Geographical Scope** | **Methods** | **Outcomes** | **Study Limitations** | **Comments** |
| **Hawley et al. (2013)^37^** | Review | To review the literature on Front of Pack (FOP)  labelling and supermarket shelf-labelling system | Food labelling (FOP) |  | No restriction | A structured search of research studies on consumer use, understanding of, preference for, perception of and behaviours relating to FOP labelling and supermarket shelf-labelling systems was performed | 28 studies were included  Consumers expressed a desire for simple FOP labels  *Cross-sectional lab based design*  ´Health halo´ is a concern for labelling as consumers can perceive products as healthy and over consume. However, one study investigated the effect of the Choices logo on chocolate mousse cake. The cake was perceived as ‘less unhealthy’ but there was no difference in taste perception or cake consumption compared to not having the logo. This suggests that the logo does not promote overconsumption |  | Only one study was discussed with the effect of FOP on food consumption and this one was investigating the ‘health halo’ effect |
| **Study ID** | **Study Design** | **Study Aim** | **Policy(ies) Analysed** | **Participants & sample size** | **Geographical Scope** | **Methods** | **Outcomes** | **Study Limitations** | **Comments** |
| **Vyth et al. (2012)**^64^ | Review | To evaluate the methodological quality of current front-of-pack labelling research and discusses future research challenges | Nutrition labelling (front of pack) |  |  | Searching literature for Studies that evaluated the effectiveness of FOP labels actually in use | 31 studies were included of which 5 focused on dietary intake and health outcomes  *Real life setting*  Simple dietary education was effective in reducing sodium intake in free-living individuals  *Modelling studies*  Replacing typical daily menus by Choices menus can potentially lead to improved nutrient intakes towards recommendations  Replacing foods by Choices-compliant foods can potentially lead to improved nutrient intakes  With Choices foods available in 2007, SAFA and sugar intake can be slightly reduced | Not a systematic review  The tool used is limited in that it relies on the researcher’s knowledge and expertise to enable fair and consistent assessments to be drawn  This tool is also limited because it does not take into account the goals of different kinds of FOP labelling research: a well-performed modelling study based on theoretical assumptions receives a higher score than a reformulation study that has a weak methodology yet uses actual reformulation data |  |

Table 2f. Food Supply Chain, Trade and Investment

*Trade and market liberalisation*

| **Study ID** | **Study Design** | **Study Aim** | **Policy(ies) Analysed** | **Participants & sample size** | **Geographical Scope** | **Methods** | **Outcomes** | **Study Limitations** | **Comments** |
| --- | --- | --- | --- | --- | --- | --- | --- | --- | --- |
| **Friel et al. (2013)**^39^ | Review | To review the available evidence on the links between trade agreements, food environments and diets from an obesity and non-communicable disease (NCD) perspective | Trade and investment |  |  | Evidence on the links between trade agreements, food environments  and diet quality and the related obesity/ non NCD issues was searched for using several databases | Nine studies were included in this review  A recent economic modelling study demonstrated that liberalization of FDI through trade agreements with the US significantly increased the amount of soft drinks consumed within signatory LMICs, and consequently increased the risk of NCDs, particularly diabetes  Of key concern in the Pacific region has been the increasing import volumes of vegetable oils, margarine, butter, meat and chickens and canned meat alongside rising saturated fat intakes. For example, between 1963 and 2000, total fat supply increased in some Pacific Island countries by as much as 80% |  | No clear discussion section present  Descriptive |
| **Study ID** | **Study Design** | **Study Aim** | **Policy(ies) Analysed** | **Participants & sample size** | **Geographical Scope** | **Methods** | **Outcomes** | **Study Limitations** | **Comments** |
| **Traill et al. (2014)**^38^ |  | To describe and discuss the dietary and nutritional changes that have occurred since the 1992 International Conference on Nutrition by attempting to untangle the multitude of factors that have contributed to such changes | Food price – in particular in relation to agricultural, trade and investment policies | High, high-middle, middle low and low income countries |  | Literature on detailed global trade models to country specific descriptions of change have been utilized. The evidence obtained has been analysed through an economic lens, although the approach used for identification and assessment was much broader than the traditional neoclassical economics approach | Between 1992 and 2007 regional per capita calorie availability increased between the 100-200 calories per capita per year. In the least developed countries there has been a per capita increase of 10.4%  Consumption response to 1%  growth in per capita incomes is more than twice as large  in low-income countries than in high-income countries (0.73; 0.60 and 0.34 for the three respective income levels)  The share of processed food in food and agricultural exports grew from 54% to 69% for high-income countries and from 49% to 67% for Asia between the 1970s and 2000s  Overall, evidence indicates the price-induced impact of trade liberalization on consumption and diets has been modest  The World Bank has estimated that even complete trade liberalization would raise prices of agricultural commodities by only 5.5%, and, thus, such results as the URAA’s achievement of only partial liberalization are unlikely to have resulted in significant dietary change  Although models indicate relatively minor price changes for agricultural commodities, trade liberalization may have induced other, less quantifiable changes in food supply systems  In India, market liberalization in the mid-1990s stimulated a rapid increase in imports of low-priced vegetable oils, which corresponded with a simultaneous increase in consumption  By contributing to a reduction in the use of nontariff barriers as trade barriers and promoting confidence in import standards, public as well as private standards have effectively increased the availability and diversity of food products traded and consumed  One study simulated the removal of support to farm commodities in the United States over the 1990s and early 2000s and found a minimal impact on calorie intakes. They also found that, as US policies have become less distorted over the 1990s and 2000s and commodity prices have become less important in determining consumer prices, the ability of agricultural policy to impact calorie intake has diminished  The fact that developing countries have had much lower support levels than developed countries implies that policies have had much less impact on diets |  | Descriptive study |

*Monetary subsidies and taxes*

| **Study ID** | **Study Design** | **Study Aim** | **Policy(ies) Analysed** | **Participants & sample size** | **Geographical Scope** | **Methods** | **Outcomes** | **Study Limitations** | **Comments** |
| --- | --- | --- | --- | --- | --- | --- | --- | --- | --- |
| **Thow et al. (2010)**^16^ | Systematic review | To assess the effect of food taxes and subsidies on diet, body weight and health through a systematic review of the literature | Monetary subsidies or taxes levied on specific food products | Not available | No restriction | Searching the English and grey literature for empirical studies and modelling studies investigating the effect of a tax or subsidy on food consumption, body weight or disease.  Empirical studies were defined as assessing the effect of an actual tax, while modelling studies predicted the outcomes of potential taxes or subsidies | One study examined seven scenarios for taxing unhealthy and subsidizing healthy foods and nutrients in Denmark. Each involved the equivalent of halving value added tax (VAT) on fruit and vegetables. They concluded that subsidizing specific nutrients was more effective than subsidizing food groups. Their best revenue-neutral scenario decreased average consumption of sugar by 6.5%, fat by 2.5% and saturated fat by 3.6%, and increased consumption of fibre by 6.5%  In addition, another study predicted that increasing soft drink prices in Norway by 27%, by doubling production taxes and VAT, would reduce average consumption by 44% in heavy soft drink consumers and by 17% in light consumers | The review’s findings are limited by the high proportion of modelling studies, which are based on assumptions and subject to data limitations  Only English language studies were included and majority of evidence came from high-income countries |  |
| **Study ID** | **Study Design** | **Study Aim** | **Policy(ies) Analysed** | **Participants & sample size** | **Geographical Scope** | **Methods** | **Outcomes** | **Study Limitations** | **Comments** |
| **Galizzi (2012)**^11^ | Review of modelling studies | To assess the relative effectiveness of each type of intervention | 1. Regulation and taxation |  | US and UK |  | *Modelling study*  An increase in VAT up to 17.5% on fat foods in the US can reduce ischemic risks of 1.8-2.6% with more a 1000 lives saved a year |  | No methods section |

*Climate change*

| **Study ID** | **Study Design** | **Study Aim** | **Policy(ies) Analysed** | **Participants & sample size** | **Geographical Scope** | **Methods** | **Outcomes** | **Study Limitations** | **Comments** |
| --- | --- | --- | --- | --- | --- | --- | --- | --- | --- |
| **Lake et al. (2012)**^41^ | Review | To investigate the potential impact of climate change on food security (nutrition and food safety) and the implications for human health in developed countries |  |  | Developed countries (using the UK as a case study) | Expert input and structured literature searches were conducted and synthesized to produce overall assessments of the likely impacts of climate change on global food production and recommendations for future research and policy changes | *The likely impact of climate change on world food prices*  Several studies suggest little change, or a small reduction, in grain prices up to a rise in global temperatures of 3°C after which prices will start to rise as production falls  One example of the impact of current climate variability occurred in 2006 when extreme weather in many parts of the world, led to reductions in world cereal production. These yield reductions were partly to blame for rising global food prices  If rises in food prices occur, then individuals may shift to lower cost food items, which in turn, may have health consequences and can result in less healthy food choices  Therefore, these rises in food prices associated with climate change may reduce the nutritional quality of dietary intakes and lower the nutritional status of some groups  Rising prices could also increase the risk of obesity particularly among children, young adults, smokers, lower income groups, and frail older people who already have more marginal nutritional status and are more likely to be affected by rising prices. Such price rises raise equity concerns and are likely to exacerbate health inequalities  Shifts to low GHG diets would reduce meat and dairy consumption, resulting in public health benefits and risks. Although a recent U.K. study estimated that a 30% reduction in red meat consumption would reduce ischemic heart disease by 15% |  | Descriptive |

Table 1g. Multi-component interventions

*Food promotion and provision*

| **Study ID** | **Study Design** | **Study Aim** | **Policy(ies) Analysed** | **Participants & sample size** | **Geographical Scope** | **Methods** | **Outcomes** | **Study Limitations** | **Comments** |
| --- | --- | --- | --- | --- | --- | --- | --- | --- | --- |
| **Blanchette & Burg (2005)**^42^ | Review | To review the current literature about potential determinants of fruit and vegetable intakes and effective intervention strategies to increase the consumption of fruits and vegetables among 6–12 year old children | School & Scouts based multi-component interventions (curriculum, education, accessibility, marketing, peer modelling) | 6-12 year old children | US, Norway, Denmark and UK | The PubMed and  Psychinfo electronic literature databases were searched for papers regarding fruit and vegetable intakes and interventions strategies to increase consumption | 38 papers were included in this review  The results of the interventions showed increases from 0 to 2.54 daily fruit and vegetable consumption servings or portions, with 14 of the 15 interventions resulting in increased consumption of fruit and/or vegetables. Increases in fruit intake were more frequent and generally more substantial than increases in vegetable intake | Qualitative review and not a meta-analysis  The differences in study designs, determinants that were included, the way these determinants were measured, as well as in intervention components between the reviewed studies was too large to conduct a more formal quantitative meta-analysis |  |
|  |  |  | Multi-component school based interventions |  |  |  | 7 school based interventions are multi-component and included parental activities, school meal modification , cafeteria/ school marketing and community interventions  Results of these multi-component interventions were all positive (results ranging from +0.2 to +1.68 portions of fruit and vegetable per day) |  |  |
|  |  |  | Multi-component school based interventions |  |  |  | One intervention included fruit and vegetable subscription as well as curriculum activities, newsletters to parents and evaluated the impact of subscription with cost versus for free  Results were from no increase in total daily intake to 0.7 portion fruit/day) |  |  |
|  |  |  | Multi-component school based interventions |  |  |  | The third intervention included video (heroic) peer modelling and rewards in addition to the fruit and vegetable distribution  Results: +2.18 to +2.54 fruit and vegetable portions/day) |  |  |
|  |  |  | Multi- component scouts based intervention |  |  |  | Two interventions were Scouts-based (curriculum/badge activities, modelling through comic books, newsletters to parents), and resulted in consumption increases of 0.4 and 0.8 fruit and vegetable/day |  |  |
| **Study ID** | **Study Design** | **Study Aim** | **Policy(ies) Analysed** | **Participants & sample size** | **Geographical Scope** | **Methods** | **Outcomes** | **Study Limitations** | **Comments** |
| **Patel & Cabana (2010)**^27^ | Review | To describe the beverages currently offered within child care facilities and schools; and summarizes school and child care based interventions and policies to encourage healthy beverage intake | Healthy beverages in school and child care settings |  | US, Germany and UK |  | *Replacing SSBs with water*  Two RCT studies associated school drinking water provision and promotion with an increase in water consumption, but no change in SSB intake |  | No clear methods section/ structure |
| **Study ID** | **Study Design** | **Study Aim** | **Policy(ies) Analysed** | **Participants & sample size** | **Geographical Scope** | **Methods** | **Outcomes** | **Study Limitations** | **Comments** |
| **Epstein et al. (2012)**^45^ | Review – experimental research | To review experimental research that can provide evidence base for policy decisions that influence food purchasing, dietary intake, and health | 1. Fiscal measures; taxes, subsidies, price elasticity |  |  | Literature searches were conducted to identify original research articles that examined food and/or nonalcoholic beverage purchases as a function of price manipulation in at least one phase of their design | 24 studies met the inclusion criteria  *Experimental study*  A 20% subsidy on healthy food, together with educational materials and blood pressure readings led to a 6% increase in consumption of healthy foods and a 2% reduction of unhealthy foods. Consumption of healthy foods increased by 17% at follow-up with maintenance of the 2% decrease in unhealthy food consumption | Experimental research can have unintended effects | Most studies looked at the effect of fiscal measures on purchases (which is not an outcome for our review) |

*Labelling and voluntary reformulation (trans-fat)*

| **Study ID** | **Study Design** | **Study Aim** | **Policy(ies) Analysed** | **Participants & sample size** | **Geographical Scope** | **Methods** | **Outcomes** | **Study Limitations** | **Comments** |
| --- | --- | --- | --- | --- | --- | --- | --- | --- | --- |
| **Downs et al. (2013)**^32^ | To systematically review evidence for the effectiveness of policies, including self-regulation, aimed at reducing industrially produced trans fatty acids (TFAs) in food | Labelling and voluntary reformulation | Systematic review |  | Brazil, Canada, Costa Rica, Denmark, the Netherlands, the Republic of Korea and the United States of America | A systematic literature search was conducted using the Medline, Embase and Cinahl databases to identify peer-reviewed articles that examined the effect of a TFA policy | The effects of mandatory TFA labelling and voluntary limits were more variable and depended largely on the food category. In particular, changes in TFA levels in margarines and bakery products were smaller in countries without a national ban  In Canada, mandatory TFA labelling combined with voluntary limits was associated with a 35% reduction in TFAs in breast milk and a 30% reduction in dietary intake in the general population | All but three studies included in this review were conducted in high-income countries  Some studies in this review were limited because they used compliance with regulations or the TFA level in blood or breast milk as an intermediate outcome and did not examine TFA intake and its associated disease risk  Studies examining TFA policy interventions in a “real-world” setting may overestimate the resulting reduction in TFA levels because of sampling limitations  The studies in the review were not directly comparable | Focus is more on TFA levels in food products after reformulation than the effect on dietary intake or health outcomes |
